# Coherent population trapping by dark state formation in a carbon nanotube quantum dot

**DOI:** 10.1038/s41467-018-08112-x

**Published:** 2019-01-22

**Authors:** Andrea Donarini, Michael Niklas, Michael Schafberger, Nicola Paradiso, Christoph Strunk, Milena Grifoni

**Affiliations:** 1 Institute for Theoretical Physics, University of Regensburg, 93040 Regensburg, Germany; 2 Institute for Experimental and Applied Physics, University of Regensburg, 93040 Regensburg, Germany

## Abstract

Illumination of atoms by resonant lasers can pump electrons into a coherent superposition of hyperfine levels which can no longer absorb the light. Such superposition is known as a dark state, because fluorescent light emission is then suppressed. Here we report an all-electric analogue of this destructive interference effect in a carbon nanotube quantum dot. The dark states are a coherent superposition of valley (angular momentum) states which are decoupled from either the drain or the source leads. Their emergence is visible in asymmetric current−voltage characteristics, with missing current steps and current suppression which depend on the polarity of the applied source-drain bias. Our results demonstrate coherent-population trapping by all-electric means in an artificial atom.

## Abstract (web summary)

Transport in quantum systems is complex and can be suppressed by coherent superposition of the involved states. Here, the authors find all-electronic suppression of transport in a carbon nanotube originating from coherent population trapping and give criteria for the presence of such a dark state.

## Introduction

Coherent population trapping (CPT) occurs in three-level, Lambda-type atomic systems coupled to two quasi-resonant electromagnetic modes^1,2^. The light beams coherently excite the atom from two low-lying states to a common excited state. By proper detuning of the lasers, the system has a finite probability to decay into a coherent superposition of the low-lying states which is decoupled from the light, a so-called dark state (DS). In the stationary limit, the DS is occupied with probability one and the CPT is perfect.

Quantum dots offer the possibility to engineer artificial atoms and molecules by proper circuit design, and hence to probe CPT in effective Lambda-systems. Proposals^3–5^ have considered microwave irradiated double quantum dot analogs of the seminal experiment^6^. Since localization of the electrons in the DS also implies a vanishing current through the double quantum dot, this allows the electrical detection of CPT by recording variations of the current as the microwaves parameters are tuned.

All-electrical realizations of CPT are also desirable. Several proposals have predicted trapping in a coherent superposition of spatially localized states under appropriate bias conditions,^7–11^ whose signatures have been observed in vertical double dot experiments^12,13^. A second possibility is, similar to the original optical experiment, CPT in a linear superposition of energy eigenstates, predicted for highly symmetric triple quantum dot setups^14,15^ or for molecular junctions with intrinsic orbital degeneracies^16–18^.

**Fig. 1:**
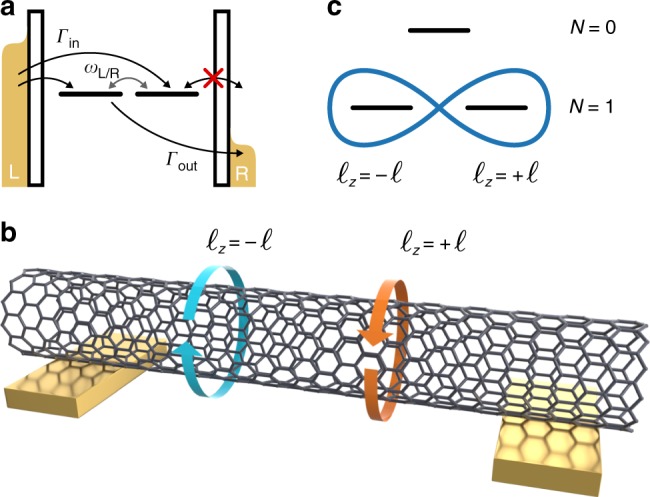
All-electronic dark states in a carbon nanotube quantum dot. **a** Quantum dot setup to probe coherent population trapping in a dark state (DS). For the chosen bias voltage polarity, an electron can enter a DS from the left lead but it cannot leave it by tunneling to the right lead. Precession allows population transfer from the dark state to the coupled state (CS). **b** A CNT features degenerate angular momentum states. Linear combinations of angular momentum states for single-electron occupancy (*N* = 1) of a given nanotube shell can give rise to CS and DS. The latter is represented by the eight-shaped orbital in panel (**c**). **c** Effective Lambda-system for coherent population trapping in a CNT quantum dot. Because an electron in the DS cannot leave the carbon nanotube, the state *N* = 0, corresponding to an empty shell configuration, can no longer be reached. CNT carbon nanotube

In this work we report the experimental realization of the second all-electrical realization of CPT in a single carbon nanotube (CNT) quantum dot. The effect requires the presence of quasi orbitally degenerate states which can form coherent superpositions. Under given conditions, tunneling events into and out of the dot successively trap the system in a DS, i.e., a coherent superposition of the degenerate levels which is decoupled from one of the leads. This in turn yields a characteristic current suppression as the bias voltage or the gate voltage are tuned. The situation is illustrated in the quantum dot setup of Fig. 1a for the case of a positive electrochemical potential drop between left and right leads. The coherent superposition of two degenerate states results in a coupled state (CS) and a DS which is decoupled from the right lead. This allows electrons to enter the DS from the left while preventing them to leave it to any of the two leads. CPT occurs and current is suppressed. For opposite bias no suppression takes place. There is a close analogy between the experiment generating dark states in atoms and the one discussed here: In both cases the coupling to an external drive (the laser fields for the atom, the DC bias for the quantum dot) enables inelastic transitions among three energy eigenstates of the isolated system. The spontaneous formation of a coherent superposition of system eigenstates (the dark state) suppresses these transitions, despite the presence of the driving. Here we demonstrate that such a situation has been realized in a CNT-based quantum dot. We notice that while in the optical setup it is the coherent drive that defines the linear combination yielding the DS^1^, in the transport setup the effect is more subtle. As seen in Eq. (2) below, the linear combination is set by the phase of the tunneling matrices which couple the quantum dot to the leads.

## Results

### Dark states in CNTs

Similar to graphene, CNTs posses an orbital (valley) degree of freedom, arising from the two inequivalent Dirac points *K*, *K*′ in the Brillouin zone. In CNTs of the zig-zag class, such orbital degree of freedom corresponds to the longitudinal orbital momentum $\hbar \ell _z$, accounting for clockwise $\left( {\ell _z = - \ell } \right)$ or anticlockwise $\left( {\ell _z = \ell } \right)$ rotations along the tube waist^19^ (see Fig. 1b). The finite length of the CNT quantum dot results in discrete longitudinal momentum states, with quantum number *m*, and hence in a shell structure like for the atomic bound states. In the absence of spin−orbit coupling^20–22^ and valley mixing^21,23–25^, each shell consists of four degenerate bound states with spin, *σ* = ↑, ↓, and angular momentum, $\ell _z = \pm \ell$, degrees of freedom. Thus each shell can accommodate up to *N* = 4 electrons. As shown in the following, for a CNT quantum dot near the *N* = 0 ↔ *N* = 1 resonance, a coherent superposition of angular momentum states can form which is decoupled from one of the two leads, and hence is a DS for the quantum dot for appropriate polarity of the applied bias voltage (see Fig. 1c). Due to the particle-hole symmetry of the many-body spectrum, CPT is expected near the *N* = 3 ↔ *N* = 4 resonance if the voltage polarity is reversed.

### Experimental signatures of CPT

**Fig. 2:**
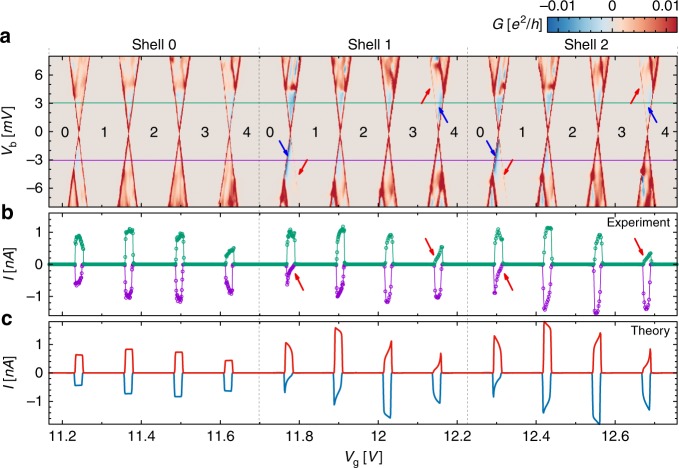
Experimental signatures of coherent population trapping. **a** Experimental differential conductance as a function of back-gate voltage *V*_g_ and bias voltage *V*_b_. Twelve consecutive Coulomb diamonds can be assigned to three carbon nanotube shells which get progressively occupied with *N* = 1 to *N* = 4 electrons. Current suppression (negative differential conductance) and faint Coulomb diamond borders are observed, indicated by blue and red arrows, respectively. **b** Current vs. gate voltage for the two values *V*_b_ = ±3.045 mV of the bias voltages corresponding to the green/purple lines in panel (**a**). Solid lines provide here a guide to the eye. Current suppression associated to coherent population trapping is indicated by red arrows. **c** Numerically evaluated stationary current qualitatively reproducing the experiment. The parameters used in the simulation are shown in Table 1

Measurements are performed on a suspended CNT grown on top of prepatterned leads. Such CNTs are usually called ultraclean owing to their low level of impurities^26^. In Fig. 2a we show the experimentally measured differential conductance *G* of our ultraclean CNT quantum dot as a function of the applied bias voltage *V*_b_ and of a back-gate voltage *V*_g_. Coulomb diamonds are clearly visible, with a characteristic fourfold periodicity, a signature of the successive filling of CNT shells with four electrons each. Noticeably, three almost identical diamonds are followed by a larger one. The width of a Coulomb diamond is a measure of the energy required to fill the CNT with an extra electron, which accounts for charging effects and single-particle properties. For the small diamonds only a charging energy has to be paid, which indicates almost degenerate states within a shell (negligibly small spin−orbit coupling and valley mixing), as well as a small exchange energy for the middle diamond. For the larger diamonds the shell is full (*N* = 0,4,8,…), such that filling the CNT with an extra electron requires to pay a charging energy and the mean inter-shell spacing. By closer inspection of the current voltage characteristics in shell 1 and shell 2, we observe signatures of current suppression in the form of faint Coulomb diamond edges and negative differential conductance (NDC) for the 0 ↔ 1 transition, as indicated by the red and blue arrows, respectively. The same pattern occurs also at the 3 ↔ 4 transition for opposite bias polarity.

**Fig. 3:**
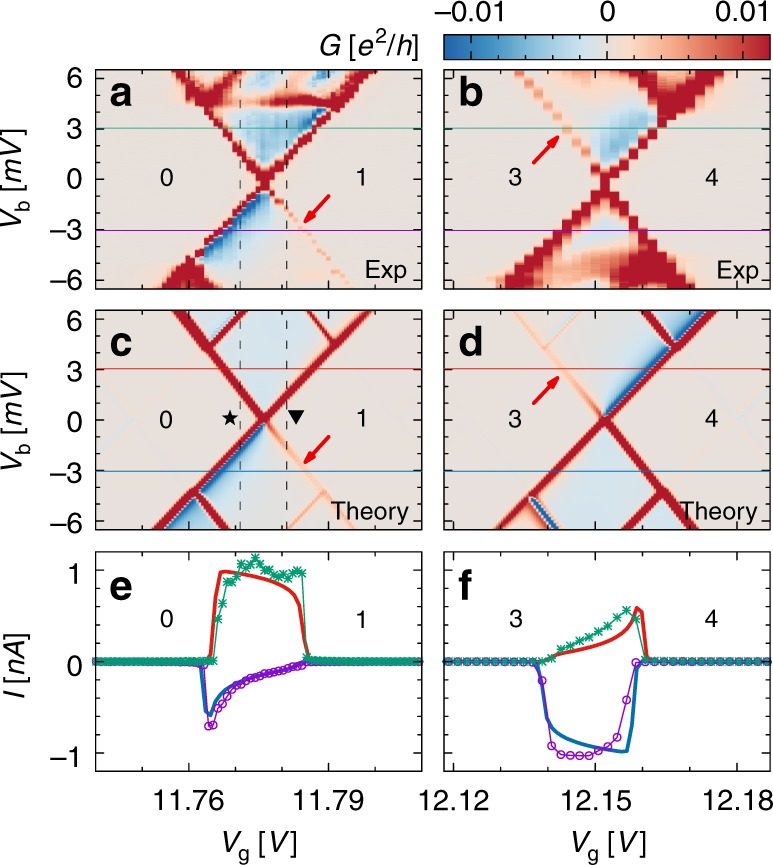
Current suppression and particle-hole symmetry. **a**, **b** Experimental stability diagrams for shell 1. Current suppression features observed for single-electron tunneling in panel (b), also occur for single-hole tunneling under reverted bias polarity and mirroring of the gate voltage, as shown in panel (**b**). **c**, **d** Theoretical stability diagrams for the 0 ↔ 1 and 3 ↔ 4 dynamical regimes reproducing the experimental observation. **e**, **f** Comparison of experimental (data points with solid guiding lines) and numerical (solid lines) current-gate traces at bias voltage set to *V*_b_ = ± 3.045 mV. The green and red (blue and purple) traces correspond to the positive (negative) bias voltage

The most frequent reason for NDC is weakly conducting (slow) excited states, resulting from strong asymmetries in the tunneling coupling of the system to the leads. This mechanism is improbable here, as it implies very different amplitudes in the coupling between time reversal partners (the different valley states) and the same lead. The strong correlation of the NDC with the conductance suppression at the edges of both the *N* + 1 and *N* + 3 Coulomb diamonds, and the absence of the latter in the *N* + 2 diamonds, suggests that CPT with formation of electronic dark state provides the correct interpretation of our experiments. To support such a statement, we have compared experimental gate traces and bias traces with theoretical calculations for the stationary current of a CNT. Figure 2b shows experimental gate traces for *V*_b_ = ±3.045 mV (green/purple line in Fig. 2a); the numerical calculations are depicted in Fig. 2c. The parameters used can be found in Table 1. The same outcome is seen in the theoretical traces. For further inspection, we have focused on the 0 ↔ 1 and 3 ↔ 4 transitions of shell 1. The respective experimental stability diagrams are shown on an enlarged gate voltage scale in Fig. 3a, b, while the corresponding numerical results are depicted in Fig. 3c, d. We observe similar behavior as in the experiment within a range of 3 mV around zero bias. At larger voltages, besides additional diagonal lines due to excited states, the experimental curves display a horizontal line not understood at present. Experimental and theoretical gate traces at fixed *V*_b_ are shown together in Fig. 3e, f.

**Table 1:** Parameters

Parameter	Shell 0	Shell 1	Shell 2
*ε*_0_		4.35 meV	
*U*		20 meV	
*J*		10 μeV	
*k*_B_ *T*		50 μeV	
*ħΓ*_R_	2 μeV	10 μeV	10 μeV
*ħΓ*_L_		4 μeV	
*ħΓ*_rel_		0.1 μeV	
Δ*ϕ*	0.01*π*	0.11*π*	0.07*π*
*η*		0.55	

Numerical parameters to fit all three shells of the experiment. Only *Γ*_L_ and Δ*ϕ* vary with the shell; all other parameters are the same for all shells

**Fig. 4:**
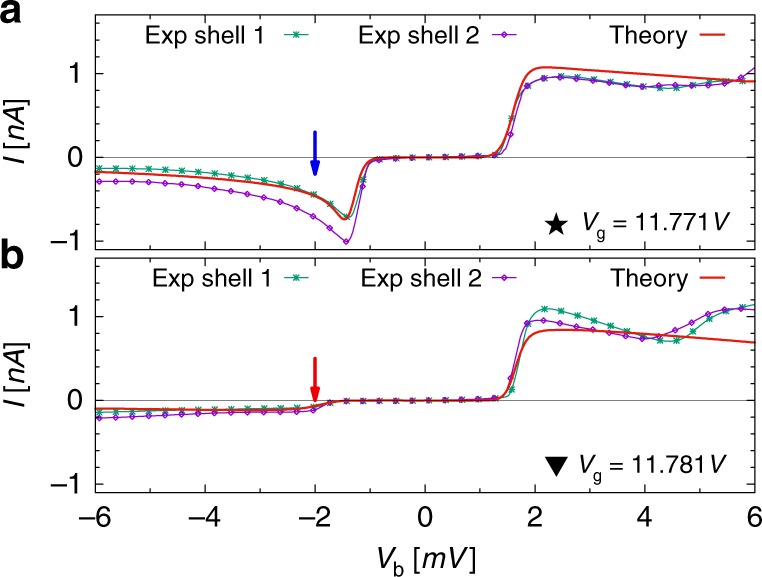
*I*−*V* characteristics in the presence of dark states**. a**, **b** Experimental current-bias characteristics (data points with solid guiding lines) around the 0 ↔ 1 resonance for shell 1 at voltages *V*_g_ = 11.771V (**a**) and *V*_g_ = 11.781 V (**b**) are compared to numerical results (thicker solid lines). These gate voltages correspond to the vertical dashed lines in Fig. 3c with the associated star and triangle labels. The behavior at positive voltages is similar. At negative bias, however, one observes a pronounced negative differential conductance in panel (**a**) and almost vanishing current in panel (**b**). The more effective coherent population trapping in (**b**) indicates a vanishing precession between the dark and the coupled state; see Fig. 1a. The current measured in shell 2 displays similar behavior

At the 0 ↔ 1 resonance both the experimental and theoretical gate traces show a rectangular shaped current at positive bias, typical of quantum dot behavior in the sequential tunneling regime; at negative bias, however, the current first increases and then gradually decreases as the gate increases, indicating trapping of a single electron. At the 3 ↔ 4 resonance similar current shapes are observed for opposite bias voltage polarity and upon gate voltage mirroring, a signature of trapping of a single hole. The dependence of the current on the bias voltage is analyzed in more detail in Fig. 4. We show the current for the 0 ↔ 1 transition at *V*_g_ = 11.771 V and *V*_g_ = 11.781 V in Fig. 4a, b, respectively; these positions are marked in Fig. 3c by vertical lines and the corresponding symbols. The NDC and the faint (almost missing) resonant line are highlighted by a blue and red arrow, respectively.

Again, the agreement between theory and experiment is remarkable. As discussed below, all the characteristic features observed in Figs. 2–4 can be explained in terms of CPT in a DS, combined with a precessional motion which transfers population between the dark and the coupled state, as sketched in Fig. 1a.

### Orbital degeneracy and CNT spectrum

**Fig. 5:**
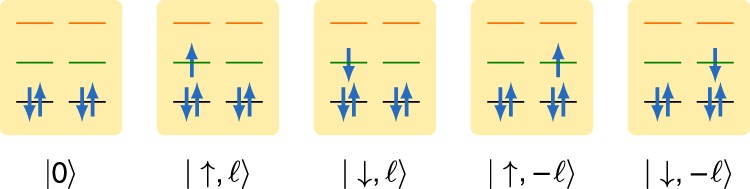
Many-body groundstates. Configuration corresponding to the *N* = 0 groundstate and to the four *N* = 1 groundstates with one extra electron in shell *m* = 1. The shells denoted in the text with *m* = 0, 1, 2 are depicted here in black, green and orange, respectively

The general ingredients to describe charge transport across a quantum dot in the sequential tunneling regime are a tunneling Hamiltonian coupling the dot to the electrodes, and the electronic spectrum of the isolated system in the energy range set by the electrochemical potentials of the lead electrodes. The presence of orbital degeneracies (or quasi-degeneracies) is decisive for the occurrence of CPT. As discussed above, the single particle energy spectrum of a CNT of finite length is fully characterized by a shell quantum number *m* and the pair $(\sigma ,\ell _z)$, accounting for the spin and orbital degrees of freedom. Curvature-induced spin−orbit coupling and valley mixing remove the intra-shell degeneracy. The amplitude of the spin−orbit coupling is largest near the Dirac point and of the order of a fraction of meV. However, it strongly decreases for states away from the bottom of the CNT conduction band^23^, which is the case for the gate voltage range in which CPT is seen in our experiment. Similarly, valley mixing due to disorder is strongly suppressed in ultraclean CNTs, and is forbidden by symmetry in CNTs of the zig-zag class^25^, which suggests that we have measured such kind of tube in our experiment. We neglect both perturbations in the following. The Hamiltonian we used to describe the CNT quantum dot is thus given in Eq. (5) and it considers three shells only *m* = 0, 1, 2. The only relevant parameters of the Hamiltonian are the inter-shell spacing *ε*_0_, the interaction *U* accounting for charging effects and the exchange interaction *J*. A many-body state with *N* particles is thus characterized by its total energy *E*, the total angular momentum *L*_*z*_, and the total spin quantum numbers *S* and *S*_*z*_, i.e., it has the form |*N*, *E*; *S*, *S*_*z*_, *L*_*z*_〉. In our three-shell model, we have fixed the energy *E*_0_ and the particle number of the configuration with the shell *m* = 0 completely full, and the upper two shells *m* = 1, 2 completely empty. The *N* = 0 groundstate |0, *E*_0_; 0, 0, 0〉 ≡ |0〉 is depicted in Fig. 5. The *N* = 1 groundstate is fourfold degenerate. A basis is the quadruplet of states $\left\{ {\left| {1,E_1;{\textstyle{1 \over 2}},\sigma ,\ell _z} \right\rangle } \right\}$ obtained by adding one electron with quantum numbers $(\sigma ,\ell _z)$ on shell *m* = 1. These states are also graphically shown in Fig. 5, where we used the short-cut notation $|\sigma ,\ell _z\rangle$. Further examples of many-body states with *N* = 1, 2 electrons are in Supplementary Note 1. Here we exemplarily focus on CPT at the 0 ↔ 1 resonance, which involves the *N* = 0 and the *N* = 1 groundstates.

### Tunneling matrix

**Fig. 6:**
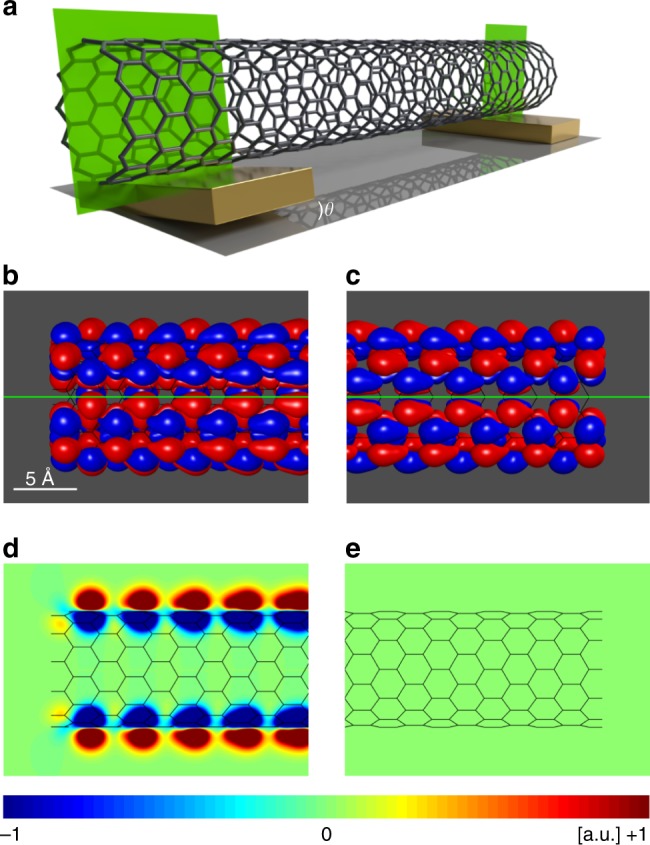
Dark state of a (12,0) carbon nanotube. **a** Sketch of the nanotube-lead configuration at the left and right contacts. The left lead is rotated by an angle *θ* with respect to the right lead. The intersection of the green rectangles with the leads define in both leads the contact region. **b**, **c** Equiamplitude surfaces of the carbon nanotube wave function as seen from the left (**b**) and right (**c**) contact region. At the left lead, a nodal line is seen that coincides with the green contact line. At the right lead, in contrast, the nodal line and the contact line do not coincide. **d**, **e** Projection of the wave function amplitudes on the intersection rectangle. The dark state has a vanishing amplitude at the right lead but not at the left lead

The tunneling Hamiltonian allows for transitions between dot states with different particle number. Its form, given in Eq. (6), is rather standard. The associated complex tunneling amplitude $t_{\alpha {\mathbf{k}}m\ell _z}$ accounts for the overlap between an electron wave function in lead *α*, characterized by the momentum **k**, and a CNT wave function for shell *m* and angular momentum $\ell _z$ in the contact region. We assume that, due to the nanotube curvature, tunneling is local and occurs only through those CNTs atoms closest to the leads. In this case, as discussed in the Methods, the rate matrix ${\mathbf {\Gamma }}_\alpha ^m$, defined element-wise by ${\it \Gamma}_{\alpha \ell _z\ell _z^\prime}^m\,(\Delta E): = \mathop {\sum}\nolimits_{\mathbf k}\, t_{\alpha {\mathbf k}m\ell _z}^ \ast t_{\alpha {\mathbf k}m\ell _z^\prime}\,\delta (\varepsilon _{\mathbf k} - {\mathrm{\Delta }}E),$ is in general nondiagonal in the angular momentum basis. Figure 6 shows the situation in which a finite amount of CNT atoms is locally contacted to the leads. In a realistic setup the leads are not flat and only few CNT atoms have a relevant tunneling coupling. For a single atom contact, or in the more general surface Γ-point approximation discussed in the Supplementary Note 3, it takes the simple form ${\it{\Gamma }}_{\alpha \ell _z\ell _z^\prime }^m = {\it{\Gamma }}_\alpha ^m{\cal R}_{\alpha \ell _z\ell _z^\prime }^m = {\it{\Gamma }}_\alpha ^me^{i\phi _\alpha ^m(\ell _z - \ell _z^\prime )}$, where the phase $\phi _\alpha ^m$ describes a global property of contact *α* for shell *m*. Both approximations can be gradually relaxed giving rise to an attenuation of the destructive interference.

### Dynamics and coherent population trapping

The dynamical quantity of interest here is the stationary current *I*. It follows from the stationary reduced density operator $\hat \rho ^\infty = \lim _{t \to \infty }\hat \rho (t)$ and the current operator $\hat I_\alpha$ at lead *α* according to $I: = I_{\mathrm{L}} = {\mathrm{Tr}}_{{\mathrm{CNT}}}\{ \hat I_{\mathrm{L}}\hat \rho ^\infty \} = - I_{\mathrm{R}}$. Notice that this requires to take the trace over the full spectrum of the isolated CNT. For weak tunneling coupling, the dynamical equations for $\hat \rho$ are easily obtained from the Liouville−von Neumann equation for the total density operator by treating the tunneling Hamiltonian as a perturbation^16,17^. For general values of the gate and bias voltages such equations have to be solved numerically. Analytical solutions are possible when the system is tuned near a transition involving only *N* and *N* + 1 particles groundstates, which is the case of interest here.

Let us consider the 0 ↔ 1 transition. In the one-dimensional *N* = 0 subspace the density matrix is a number, *ρ*_0_. In the four-dimensional *N* = 1 subspace, it is block-diagonal in spin (since spin is conserved during tunneling) but not in angular momentum. The contributions from different spin configurations can be summed up in the dynamical equations yielding a set of coupled equations for *ρ*_0_ and a 2 × 2 matrix *ρ*_1_^15^. Away from the exact resonance (i.e. from the border of the Coulomb diamond), one finds for positive electrochemical potential drop $eV_{\mathrm{b}} \gg k_{\mathrm{B}}T$,

1 $$\begin{array}{*{20}{l}} {\dot {\boldsymbol\rho} _0} \hfill & = \hfill & { - \frac{i}{\hbar }[{\mathbf{H}}_{{\mathrm{LS}}},{\boldsymbol\rho} _1] + 2{\it{\Gamma }}_{\mathrm{L}}{\cal R}_{\mathrm{L}}\rho _0 - \frac{{{\it{\Gamma }}_{\mathrm{R}}}}{2}\{ {\cal R}_{\mathrm{R}},{\boldsymbol\rho}_1\} ,} \hfill \\ {\dot \rho _0} \hfill & = \hfill & {{\it{\Gamma }}_{\mathrm{R}}{\mathrm{Tr}}\left( {{\cal R}_{\mathrm{R}}{\boldsymbol\rho}_1} \right) - 4{\it{\Gamma }}_{\mathrm{L}}\rho _0,} \hfill \end{array}$$

with ${\it{\Gamma }}_{\alpha} ^{m} = {\it{\Gamma }}_{\alpha}$ and the coherence matrices ${\cal R}_{\alpha}$. Equation (1) describes relaxation governed by the terms ${\it{\Gamma }}_{\alpha} {{\cal R}}_{\alpha}$ as well as precession through the Lamb Shift contribution ${\mathbf{H}}_{{\mathrm{LS}}} = \frac{\hbar }{2}\mathop {\sum}\nolimits_{\alpha} \omega _{\alpha} {{\cal R}}_{\alpha}$. The latter originates from virtual processes from the system to the leads^14,18,27^ and its effect will be discussed later. Due to the nondiagonal form of the ${\cal R}_\alpha$ in the angular momentum basis, also the stationary density matrix ${\rho}_{1}^{\infty}$ is not diagonal there. Its diagonalization yields the stationary eigenstates, and the associated eigenvalues define the occupation probabilities of the eigenstates. To proceed, we assume for the coherence matrices the simple form ${\cal R}_{\alpha \ell _z\ell _z^\prime } = e^{i\phi _\alpha (\ell _z - \ell _z^\prime )}$ and *ϕ*_R_ ≠ *ϕ*_L_. In this case, eigenstates of ${\boldsymbol\rho}_{1}^{\infty}$ are a decoupled state (DS) at the right lead and its associated orthogonal coupled state (CS), with eigenvalues 1 and 0, respectively. Hence, Eq. (1) predicts CPT with probability one in the DS, and consequently a vanishing stationary current (see Fig. 1a). Accounting also for the spin degree of freedom, the dark/coupled states at the right lead are the linear combinations

2 $$\left\{ {\begin{array}{*{20}{c}} {|{\mathrm{DS}},\sigma ;{\mathrm{R}}\rangle } \\ {|{\mathrm{CS}},\sigma ;{\mathrm{R}}\rangle } \end{array}} \right. \equiv \frac{1}{{\sqrt 2 }}\left( {e^{i\ell \phi _{\mathrm{R}}}|\sigma ,\ell \rangle \mp e^{ - i\ell \phi _{\mathrm{R}}}|\sigma, - \ell \rangle } \right),$$

where R indicates the right lead. Equation (2) also implies that |DS, *σ*; R〉 satisfies the equation

3 $$\langle 0|\hat d_{{\mathrm{R}}\sigma }|{\mathrm{DS}},\sigma ;{\mathrm{R}}\rangle = 0,$$

where $\hat d_{{\mathrm{R}}\sigma } = {\textstyle{1 \over {\sqrt 2 }}}(e^{i\ell \phi _{\mathrm{R}}}\hat d_{\ell \sigma } + e^{ - i\ell \phi _{\mathrm{R}}}\hat d_{ - \ell \sigma })$ is the electron annihilator involved in the local tunneling process at the right lead. Notice the explicit dependence on the tunneling phase acquired upon tunneling at the lead R. If *ϕ*_R_ = *ϕ*_L_, the DS in Eq. (2) is also decoupled at lead L; transport solely occurs through the CS and CPT cannot occur. Thus CPT requires *ϕ*_R_ ≠ *ϕ*_L_.

The DS wave function in Eq. (2) is explicitly shown in Fig. 6 on the example of a (12, 0) CNT. We have assumed the angular coordinate of the contact atoms at the right and left lead to be rotated by a small angle *θ* = *π*/24. The DS has a node at the contact positions at the right lead but not at the left lead. The corresponding CS is shown in Supplementary Fig. 1 and has finite weight at both contacts.

Let us now turn to the impact of the Lamb shift term in Eq. (1). It introduces a precession of the Bloch vector in the CS/DS basis of Eq. (2) with population transfer between dark and coupled states. The frequencies *ω*_L_, *ω*_R_ are given in Eq. (11). For the situation indicated in Fig. 1a, *ω*_L_ ≠ 0 allows the electrons in a DS to precess into the coupled state and from there to escape, yielding a small stationary current $I = 4e{\it{\Gamma }}_{\mathrm{L}}\rho _0^\infty$. We find from Eq. (1) the expression

4 $$I = \frac{{4e{\it{\Gamma }}_{\mathrm{R}}\omega _{\mathrm{L}}^2{\mathrm{cos}}^2{\mathrm{\Delta }}\phi }}{{2{\it{\Gamma }}_{\mathrm{R}}^2 + 2(\omega _{\mathrm{L}} - \omega _{\mathrm{R}})^2 + \omega _{\mathrm{L}}\left( {\omega _{\mathrm{L}}{\it{\Gamma }}_{\mathrm{R}}/{\it{\Gamma }}_{\mathrm{L}} + 4\omega _{\mathrm{R}}} \right){\mathrm{cos}}^2{\mathrm{\Delta }}\phi }},$$

for Δ*ϕ* = (*ϕ*_L_ − *ϕ*_R_)/2 ≠ 0. Because the precession frequencies strongly depend on gate and bias voltage, also the effectiveness of CPT does, as clearly seen from the gradual variation of the current in Fig. 4. Here, the traces in panels (a) and (b) correspond to two distinct values of *V*_g_. Clearly, at negative bias voltages (corresponding to positive *eV*_b_), CPT is more pronounced in panel (b) than in panel (a), indicating a smaller *ω*_L_. As the bias polarity is changed, also the role of the precession frequencies is exchanged. The fact that at positive bias the current shows standard Coulomb steps, giving rise to rectification, is because of large *ω*_R_ (compared to *ω*_L_) for both of the chosen *V*_g_ values. This rectification is a consequence of the asymmetry in the coupling to the leads. Equal couplings would restore the left−right symmetry, the rectification would vanish but the interference blocking would remain. The dependence of the frequencies *ω*_L/R_ on *V*_b_ for the same parameters used in the simulation of Fig. 4a is explicitly shown in Supplementary Fig. 2. In Supplementary Fig. 3, we additionally show the dependence of the current when additional inelastic relaxation processes are added in the numerical simulations.

We observe that, due to the particle-hole symmetry of the spectrum with respect to half-filling (shell with *N* = 2), the equations for the reduced density operator near the 3 ↔ 4 transition immediately follow from Eq. (1) upon exchanging R with L and *V*_b_ with −*V*_b_. This implies that the CPT features at the 3 ↔ 4 transition can be obtained from the ones at the 0 ↔ 1 resonance by mirroring of both *V*_b_ and *V*_g_. This is clearly observed in Fig. 3.

Similar to the *N* = 0 groundstate, also the *N* = 2 groundstate is a singlet for positive exchange interaction *J* (see Eq. (7) of the Supplementary Note 2). However, CPT is not seen at the 1 ↔ 2 and 2 ↔ 3 resonances. Whether or not interference occurs depends on the strength of the exchange coupling, since *J*/2 is the separation to the first excited doublet of states which do not form dark states. For $J \simeq \hbar {\it{\Gamma }} \simeq eV_{\mathrm{b}}$ the excited doublet is soon into the transport window and no CPT is seen. The *N* = 2 states and a special realization of a DS for the case *J* = 0 are further discussed in Supplementary Note 1.

## Discussion

The results presented so far show a remarkable quantitative agreement between the experimental data and the theoretical predictions, strongly supporting the claim that the observed current suppression features are due to CPT. A natural question is how robust CPT is, and under which conditions can it be observed in other CNT-based quantum dots. According to our model, the effect is quite generic, as the main requirements are: first, the presence of a symmetry ${\cal S}$ of the system yielding degenerate energy states (for weak symmetry breaking the level splitting should be smaller than the tunneling broadening *Γ* = *Γ*_L_ + *Γ*_R_); second, the tunneling matrices being not diagonal in the basis associated to the symmetry ${\cal S}$ and with modulus of the off-diagonal elements of the coherence matrices ${\cal R}_\alpha$ close to one; third, the strong Coulomb interaction enforcing single electron tunneling. The requirements above are simultaneously met for *n*-doped (electron conduction) CNTs of the zig-zag class, whose bound states have angular momentum (valley) degeneracy. For this nanotube class, symmetry breaking perturbations are spin−orbit coupling and valley mixing. While the former is an intrinsic property of the CNT and decreases away from the band gap, valley mixing is due to disorder or to perturbations which break the rotational symmetry. Suspended ultraclean CNTs^26^ show very weak disorder and symmetry breaking mostly occurs due to the presence of contact leads in the nonsuspended portion of the tube. In a realistic CNT-device the curvature of the nanotube and some roughness of the contacts causes tunneling to occur locally through few single carbon atoms. This ensures on the one hand that the tunneling matrix is not diagonal in the angular momentum basis, and on the other that the tunneling is a small perturbation and hence that valley mixing is small. Furthermore, weak tunneling makes it easier to reach the sequential tunneling regime, which is typically observed for CNTs in the electron conduction regime^19^. We notice that the second requirement among the ones listed above rules out the possibility that the excited states blocking recently reported in a CNT-based quantum dot with broken fourfold degeneracy is due to the CPT discussed here^28^. While the three above-mentioned properties provide a sufficient criterion for the presence of dark states, they are not intended as a necessary one. There are indeed various setups where transport can be suppressed due to the formation of a coherent superposition of quantum mechanical states. However, it is quite different whether the coherent superposition occurs among localized position eigenstates^7,12,13^, or degenerate energy eigenstates as in our setup. For the system proposed in ref. ^7^, the DS is an energy eigenstate of the isolated system whose formation does not require phase coherence during the tunneling process. We also comment on the interference phenomena observed in the linear conductance of molecular junctions in the strong coupling regime^29,30^. Since energy conservation is required only within an accuracy set by ${\mathrm{\Delta }}E \approx \hbar {\it{\Gamma }}$, in the strong coupling regime, where *Γ* is the largest scale in the problem, the CPT based on linear superpositions of quasi-degenerate states of the isolated system loses its significance. Rather, interference based on a coherent superposition of different trajectories along the molecule becomes possible^31^. Similarly, the zero-bias interference discussed for off-resonant transport through single molecules^32^ require energy-nonconserving cotunneling transitions, and are thus distinct from the interference blockade phenomena presented in this work. We finally comment on the canyon of conductance observed in an InSb nanowire quantum dot in magnetic field^33^. In this experiment, the conductance suppression is visible throughout the entire gate voltage−magnetic field region and it encompasses both the cotunneling as well as the sequential tunneling regime. As a consequence of the strong coupling of the degenerate levels to the leads, *Γ* > *k*_B_*T*, higher order virtual processes (charge fluctuations) strongly influence the position of the conductance canyon, especially in the sequential tunneling regime. A detailed study of current suppression accounting for level broadening in the framework of the so-called 2vN approximation is found in ref. ^34^.

In conclusion, we have demonstrated experimentally and theoretically that CPT with dark state formation can be realized in a CNT quantum dot by all-electrical means. In the case considered here, the orbital degeneracies result from the interplay of the tubular nanotube geometry and the underlying graphene honeycomb lattice. However, the phenomenon is rather generic and is expected to occur in other highly symmetric quantum dot systems, e.g. in organic molecules in scanning tunneling microscope setups^35^, or in symmetric triangular quantum dots^15^, in the weak tunneling regime. In other setups degeneracies can be tuned by an external magnetic field^12,13^. Trapping in a dark state can be achieved by simple tuning of the bias or gate voltage.

## Methods

### Sample details

On a highly p-doped Si substrate with a 500 nm thermally grown SiO_2_ layer, ring-like electrode structures were defined by electron beam lithography and evaporation of 20 nm Re and 40 nm Co. A catalyst for the CVD process was deposited in the center of the electrode ring structure to increase the chance of a CNT connecting the contacts. The CNT growth was performed as the last sample fabrication step, a procedure that is aimed at yielding ultraclean devices^26^. Neither magnetoresistance nor magneto-optical Kerr effect (MOKE) measurements showed magnetic behavior of the Re/Co contacts after CVD, ruling out spin valve effects with a similar *I*–*V* characteristics^27^. The current as well as the differential conductance were measured using an Ithaco DL1211 *I*–*V*-converter.

### Model

The single-electron transistor setup is modeled by the total Hamiltonian $\hat H = \hat H_{{\mathrm{CNT}}} + \hat H_{{\mathrm{leads}}} + \hat H_{{\mathrm{tun}}}$, describing the CNT quantum dot (QD) weakly coupled to leads by a tunneling Hamiltonian $\hat H_{{\mathrm{tun}}}$.

The CNT-QD consists of a set of longitudinal modes, called shells, with shell index *m*. The electrons have both spin *σ* ∈ {↑, ↓} and valley $\ell _z \in \{ \ell , - \ell \}$ degrees of freedom and, since spin−orbit coupling and valley mixing are negligible in the experiment, the single-particle spectrum of a single shell is assumed to be fourfold degenerate. We include a charging term *U* and the exchange interaction *J*. The latter strongly depends on the CNT chirality and radius and was assumed to be 10 μeV. The CNT-QD Hamiltonian reads^19,36^

5 $$\begin{array}{*{20}{l}} {\hat H_{{\mathrm{CNT}}}} \hfill & = \hfill & {\mathop {\sum}\limits_{m\ell _z} \left( {m\varepsilon _0 - e\alpha _{\mathrm{g}}V_{\mathrm{g}}} \right)\hat n_{m\ell _z} + \frac{U}{2}\hat N^2} \hfill \\ {} \hfill & {} \hfill & { + J\mathop {\sum}\limits_m \left( {{\hat{\mathbf S}}_{m\ell } \cdot {\hat{\mathbf S}}_{m - \ell } + \frac{1}{4}\hat n_{m\ell }\hat n_{m - \ell }} \right),} \hfill \end{array}$$

where in the numerical calculations only three shells (*m* ∈ {0, 1, 2}) are considered (see Fig. 5). The gate voltage *V*_g_ applied with a level arm *α*_g_ ensures particle-hole symmetry of Eq. (5) with respect to shell *m* = 1 for *eα*_g_*V*_g_ = *ε*_0_ + 6*U* + *J*/4. Further, the occupation operator $\hat n_{m\ell _z} = \mathop {\sum}\nolimits_\sigma \hat d_{m\ell _z\sigma }^\dagger \hat d_{m\ell _z\sigma }$, and the spin operator $\widehat {\mathbf{S}}_{m\ell _z} = \frac{1}{2}\mathop {\sum}\nolimits_{\sigma \sigma \prime } \hat d_{m\ell _z\sigma }^\dagger {\boldsymbol{s}}_{\sigma \sigma \prime }\hat d_{m\ell _z\sigma \prime }$ are defined in terms of annihilation (creation) operators $\hat d_{m\ell _z\sigma }^{(\dagger )}$ of an electron in shell *m* with angular momentum $\ell _z$ and spin projection *σ*.

The CNT Hamiltonian can be diagonalized analytically by using the basis corresponding to the eigenstates of the total particle number $\hat N = \mathop {\sum}\nolimits_{m\ell _z} \hat n_{m\ell _z}$, total spin $\hat S^2 = \mathop {\sum}\nolimits_{m\ell _z} {\hat{\mathbf S}}_{m\ell _z}^2$, total spin projection $\hat S_z = \frac{1}{2}\mathop {\sum}\nolimits_{m\ell _z\sigma } \sigma \hat d_{m\ell _z\sigma }^\dagger \hat d_{m\ell _z\sigma }$, and total angular momentum operator $\hat L_z = \mathop {\sum}\nolimits_{m\ell _z} \ell _z\hat n_{m\ell _z}$. Accordingly, many-body states are uniquely defined by the vector set {|*N*, *E*; *S*, *S*_*z*_, *L*_*z*_〉}. In our three-shell model, the *N* = 0 groundstate corresponds to the shell *m* = 0 being completely full, and the *N* = 1 groundstate is given by the quadruplet $\left\{ {\left| {1,\varepsilon _0;\frac{1}{2},\sigma ,\ell _z} \right\rangle } \right\}$, as shown in Fig. 5. Examples of many-body states with occupation *N* = 2 can be found in Supplementary Note 1.

The electrons in the leads are considered as fermionic reservoirs of noninteracting electrons at temperature *T* and chemical potentials *μ*_L_ = *μ*_0_ + *eηV*_b_, *μ*_R_ = *μ*_0_ + *e*(*η* − 1)*V*_b_ for the left (L) and right (R) lead, respectively, with *V*_b_ the applied bias voltage. The parameter 0 < *η* < 1 accounts for an asymmetric bias drop at the two leads. For the investigated setup a good fit to the data was obtained for almost symmetric potential drop (*η* = 0.55). For simplicity, the underlying Hamiltonian is chosen to be the one of a noninteracting electron gas $\hat H_{{\mathrm{leads}}} = \mathop {\sum}\nolimits_{\alpha {\mathbf{k}}\sigma } \varepsilon _{\mathbf{k}}\hat c_{\alpha {\mathbf{k}}\sigma }^\dagger \hat c_{\alpha {\mathbf{k}}\sigma }$, where $\hat c_{\alpha {\mathbf{k}}\sigma }^{(\dagger )}$ annihilates (creates) an electron in lead *α* ∈ {L, R} with momentum **k** and spin projection *σ*.

The leads are weakly coupled to the CNT via the tunneling Hamiltonian

6 $$\hat H_{{\mathrm{tun}}} = \mathop {\sum}\limits_{\alpha {\mathbf{k}}m\ell _z\sigma } t_{\alpha {\mathbf{k}}m\ell _z}\hat d_{m\ell _z\sigma }^\dagger \hat c_{\alpha {\mathbf{k}}\sigma } + {\mathrm{h}}{\mathrm{.c}}.$$

Thus, $\hat H_{{\mathrm{tun}}} = \mathop {\sum}\nolimits_\alpha \hat H_{{\mathrm{tun}},\alpha }$ removes (adds) an electron from the left/right lead (*α* = L/R) with momentum **k**, energy *ε*_**k**_ and spin *σ* and it adds (removes) an electron in the dot with the same spin *σ* in the state $(m,\ell _z,\sigma )$.

The tunneling Hamiltonian Eq. (6) rules the dynamics of the coupled CNT-leads system. For weak coupling, it can be treated as a perturbation. Expanding the Liouville von Neumann equation for the total density operator up to second order in *H*_tun_, and taking the trace over the reservoirs, the equations for the reduced density operator are obtained^16,17^. They are ruled by a tunneling kernel, which, according to Eq. (6), has the form

7 $${\it{\Gamma }}_{\alpha \ell _z\ell _z^\prime }^m({\mathrm{\Delta }}E): = \frac{{2\pi }}{\hbar }\mathop {\sum}\limits_{\mathbf k} t_{\alpha {\mathbf{k}}m\ell _z}^{\ast} t_{\alpha {\mathbf{k}}m\ell _z^\prime }{\mathrm{\delta }}(\varepsilon _{\mathrm{k}} - {\mathrm{\Delta }}E),$$

and is in general nondiagonal in the angular momentum basis. Rather ${\mathbf {\Gamma }}_\alpha ^m = {\it{\Gamma }}_\alpha ^m{{\cal R}}_\alpha$, where the coherence matrices ${{\cal R}}_\alpha$ are hermitian. Explicitly, ${\cal R}_{\alpha \ell _z\ell _z} = 1$ while the off-diagonal elements satisfy $0 \le |{\cal R}_{\alpha \ell _z, - \ell _z}| \le 1$. As discussed in Supplementary Note 3, the ${{\cal R}}_\alpha$ become exactly diagonal in the limit in which all the atoms of the CNT are equally coupled to the leads. In the opposite case of a single atom contact along the CNT circumference, or in the more general so-called surface Γ-point approximation, see Supplementary Note 3, one finds the optimal coherence, and the coherence matrices at lead *α* = L,R assume the form ${\cal R}_{\alpha \ell _z\ell _z^\prime } = e^{i\phi _\alpha (\ell _z - \ell _z^\prime )}$ used in the main manuscript. Then, in the dark and coupled states basis of Eq. (2), ${{\cal R}}_{\mathrm{R}}$ becomes diagonal but not ${{\cal R}}_{\mathrm{L}}$. Explicitly,

8 $${{\cal R}}_{\mathrm{R}} = \left( {\begin{array}{*{20}{c}} 0 & 0 \\ 0 & 2 \end{array}} \right),\quad{{\cal R}}_{\mathrm{L}} = 2\left( {\begin{array}{*{20}{c}} {{\mathrm{sin}}^2{\mathrm{\Delta }}\phi } & { - i\,{\mathrm{sin}}\,{\mathrm{\Delta }}\,\phi \,{\mathrm{cos\Delta }}\phi } \\ {i\,{\mathrm{sin}}\,{\mathrm{\Delta }}\phi \,{\mathrm{cos}}\,{\mathrm{\Delta }}\phi } & {{\mathrm{cos}}^2{\mathrm{\Delta }}\phi } \end{array}} \right).$$

The good agreement found between theory and experiment suggests that in our setup the coherence matrices have off-diagonal elements with modulus close to one in the angular momentum basis, i.e. tunneling occurs only at few atomic positions where the CNT is closest to the leads.

We observe that in some nanotube-based devices the quantum dot region is defined electrostatically. This yields a strong variation of the tunneling coupling and charging energy, and in turn of the current, with the gate voltage^19^. In contrast, in our experiment we did not observe strong variations of the conductance as the gate voltage was swept from the *p*-doped region across the band gap down to the deep *n*-doped regime considered here. This supports our assumption that the bottleneck for transport is given by the contact region between the metallic leads and the CNT.

### Tunneling dynamics at the 0 ↔ 1 resonance

For the parameters used in this work, the single particle groundstate is fourfold degenerate, while the two particles groundstate is only at a distance $J/2 \approx \hbar {\it \Gamma}$ from the doublet of first excited states, as discussed in Supplementary Note 1. Thus, the secular approximation is nonvalid when transitions involving the low-lying two-particle states are considered. To account for the nonsecular contributions, the equations for the reduced density matrix have thus been derived along the lines discussed in ref. ^17^. In addition, a relaxation term was added to account for inelastic processes due to e.g. phonons. Such equations have been then solved numerically within the three-shell approximation for the CNT spectrum discussed above, and by including all excitations up to a cut-off energy 1.5*ε*_0_.

Here we focus on the dynamics near the 0 ↔ 1 resonance, where only the 0 and 1 particle groundstates play a role and an analytical treatment is possible. Furthermore, the secular approximation holds true yielding for the stationary reduced operator the equation

9 $$0 = {\cal L}\hat \rho ^\infty = - \frac{i}{\hbar }\left[ {\hat H_{{\mathrm{CNT}}} + \hat H_{{\mathrm{LS}}},\hat \rho ^\infty } \right] + {\cal L}_{{\mathrm{tun}}}\hat \rho ^\infty + {\cal L}_{{\mathrm{rel}}}\hat \rho ^\infty ,$$

where ${\cal L}$ is the Liouville superoperator that contains the system internal dynamics in a commutator structure together with the Lamb shift contribution $\hat H_{{\mathrm{LS}}}$, a tunneling part ${\cal L}_{{\mathrm{tun}}}$ and a relaxation part ${\cal L}_{{\mathrm{rel}}}$. For positive potential drop *eV*_b_, the equation of motion for the *ρ*_0_ and *ρ*_1_ subblocks, where the contribution over spin configurations has been summed, reads

10 $$\begin{array}{*{20}{l}} 0 \hfill & = \hfill & {\dot {\boldsymbol\rho} _1 = - \frac{i}{\hbar }[{\mathbf{H}}_{{\mathrm{LS}}},{\boldsymbol\rho} _1] + 2{\it{\Gamma }}_{\mathrm{L}}{\cal R}_{\mathrm{L}}\rho _0 - \frac{{{\it{\Gamma }}_{\mathrm{R}}}}{2}\{ {\cal R}_{\mathrm{R}},{\boldsymbol\rho} _1\} } \hfill \\ {} \hfill & {} \hfill & { - {\it{\Gamma }}_{{\mathrm{rel}}}[{\boldsymbol\rho} _1 - {\boldsymbol\rho} _{1,{\mathrm{th}}}{\mathrm{Tr}}({\boldsymbol\rho} _1)],} \hfill \\ 0 \hfill & = \hfill & { = \dot \rho _0 = {\it{\Gamma }}_{\mathrm{R}}{\mathrm{Tr}}\left( {{\cal R}_{\mathrm{R}}{\boldsymbol\rho} _1} \right) - 4{\it{\Gamma }}_{\mathrm{L}}\rho _0,} \hfill \end{array}$$

where *ρ*_1,th_ = 1/2 is the thermal density matrix for the one-electron subblock. The dynamics is thus fully governed by the coherence matrices ${\cal R}_\alpha$. They enter in the tunneling terms ${\it{\Gamma }}_\alpha {\cal R}_\alpha$ as well as in the angular Lamb shift Hamiltonian matrix ${\mathbf{H}}_{{\mathrm{LS}}} = \frac{\hbar }{2}\mathop {\sum}\nolimits_\alpha \omega _\alpha {\cal R}_\alpha$. The latter introduces a precession with frequencies^17^,

11 $$\omega _\alpha (V_{\mathrm{g}},V_{\mathrm{b}}) = \frac{{{\it{\Gamma }}_\alpha }}{\pi }\left[ {p_\alpha ( - eV_{\mathrm{g}}) - p_\alpha \left( {U - \frac{J}{2} - eV_{\mathrm{g}}} \right)} \right],$$

where we chose an offset in the gate voltage such that the resonance is exactly at *V*_g_ = 0. Furthermore, $p_\alpha \left( {{\mathrm{\Delta }}E} \right): = - {\mathrm{Re}}\psi \left[ {1/2 + i({\mathrm{\Delta }}E - \mu _\alpha )/2\pi k_{\mathrm{B}}T} \right],$ where *ψ* is the digamma function. These precession frequencies clearly depend on the gate voltage and, via the chemical potentials, also on the bias voltage. This dependence is further analyzed in Supplementary Note 2. Notice also the dependence of *ω*_*α*_ on the charging energy *U* and exchange interaction *J*, that results from virtual processes involving also the two electron subspaces. The above equations are general. To proceed, we assume full coherence, such that ${\cal R}_{\alpha \ell _z\ell _z^\prime } = e^{i\phi _\alpha \left( {\ell _z - \ell _z^\prime } \right)}$. Then, when expressed in matrix form, Eq. (10) yields the set of coupled equations for populations and coherences

12 $$\begin{array}{*{20}{l}} {\dot \rho _0} \hfill & = \hfill & { - 4{\it{\Gamma }}_{\mathrm{L}}\rho _0 + {\it{\Gamma }}_{\mathrm{R}}(\rho _{\ell \ell } + \rho _{ - \ell - \ell })} \hfill \\ {} \hfill & {} \hfill & { + {\it{\Gamma }}_{\mathrm{R}}(e^{ - 2i\phi_{\mathrm R}}\rho _{\ell - \ell } + e^{2i\phi_{\mathrm R}}\rho _{\ell - \ell}^ \ast ),} \hfill \\ {\dot \rho _{\ell \ell }} \hfill & = \hfill & { - {\it{\Gamma }}_{\mathrm{R}}\rho _{\ell \ell } + 2{\it{\Gamma }}_{\mathrm{L}}\rho _0 - \left( {\frac{{{\it{\Gamma }}_{\mathrm{R}}}}{2}e^{ - 2i\phi _{\mathrm R}} - i\tilde \omega ^ \ast } \right)\rho _{\ell - \ell }} \hfill \\ {} \hfill & {} \hfill & { - \left( {\frac{{{\it{\Gamma }}_{\mathrm{R}}}}{2}e^{2i\phi _{\mathrm R}} + i\tilde \omega } \right)(\rho _{\ell - \ell })^ \ast - \frac{{{\it{\Gamma }}_{{\mathrm{rel}}}}}{2}\left( {\rho _{\ell \ell } - \rho _{ - \ell - \ell }} \right),} \hfill \\ {\dot \rho _{ - \ell - \ell }} \hfill & = \hfill & { - {\it{\Gamma }}_{\mathrm{R}}\rho _{ - \ell - \ell } + 2{\it{\Gamma }}_{\mathrm{L}}\rho _0 - \left( {\frac{{{\it{\Gamma }}_{\mathrm{R}}}}{2}e^{ - 2i\phi _{\mathrm R}} - i\tilde \omega ^ \ast } \right)\rho _{\ell - \ell }} \hfill \\ {} \hfill & {} \hfill & { - \left( {\frac{{{\it{\Gamma }}_{\mathrm{R}}}}{2}e^{2i\phi _{\mathrm R}} + i\tilde \omega } \right)(\rho _{\ell - \ell })^ \ast - \frac{{{\it{\Gamma }}_{{\mathrm{rel}}}}}{2}\left( {\rho _{ - \ell - \ell } - \rho _{\ell \ell }} \right),} \hfill \\ {\dot \rho _{\ell - \ell }} \hfill & = \hfill & { - ({\it{\Gamma }}_{\mathrm{R}} + {\it{\Gamma }}_{{\mathrm{rel}}})\rho _{\ell - \ell } - \left( {\frac{{{\it{\Gamma }}_{\mathrm{R}}}}{2}e^{2i\phi _{\mathrm R}} - i\tilde \omega } \right)\rho _{\ell \ell }} \hfill \\ {} \hfill & {} \hfill & { + 2{\it{\Gamma }}_{\mathrm{L}}e^{2i\phi _{\mathrm R}}\rho _0 - \left( {\frac{{{\it{\Gamma }}_{\mathrm{R}}}}{2}e^{ - 2i\phi _{\mathrm R}} + i\tilde \omega ^ \ast } \right)\rho _{ - \ell - \ell },} \hfill \end{array}$$

with $\tilde \omega = \omega _{\mathrm{L}}e^{2i\phi _{\mathrm{L}}} + \omega _{\mathrm{R}}e^{2i\phi _{\mathrm{R}}}$. These equations can be solved in the stationary limit with $\dot \rho _0 = 0$ and $\dot \rho _{\ell ,\ell^\prime } = 0$, together with the condition ${\mathrm{Tr}}\boldsymbol{\rho} = \rho _{\ell \ell } + \rho _{ - \ell - \ell } + \rho _0 = 1$. If *ϕ*_L_ = *ϕ*_R_ and Γ_rel_ = 0 the dark state is completely decoupled from the dynamics and therefore the stationary solution is not uniquely defined but rather depends on the initial state. Any finite relaxation rate or phase difference solves this problem. Since the general expression for the current is quite lengthy, we focus on two limiting cases. For vanishing relaxation rate but Δ*ϕ* = (*ϕ*_L_ − *ϕ*_R_)/2 ≠ 0, we obtain the current $I({\it{\Gamma }}_{{\mathrm{rel}}} = 0) = 4e{\it{\Gamma }}_{\mathrm{L}}\rho _\infty ^0$ given in Eq. (4) of the main manuscript.

As mentioned before, this expression is only valid for a non-zero phase difference. For negative bias the current is the same upon exchanging L ↔ R and the overall sign. This expression shows that the current is completely suppressed for Δ*ϕ* = *π*/2 despite finite Lamb shift. The second limit that can be analyzed is a finite relaxation rate and Δ*ϕ* = 0. Interestingly, the dependence of the current on the relaxation rate drops completely and we obtain

13 $$I(\Delta \phi = 0) = e\frac{{4{\it{\Gamma }}_{\mathrm{L}}{\it{\Gamma }}_{\mathrm{R}}}}{{4{\it{\Gamma }}_{\mathrm{L}} + {\it{\Gamma }}_{\mathrm{R}}}}.$$

Notice that this same expression for the current holds true for transport through fourfold degenerate levels in the absence of interference. The behavior of the current as a function of the phase difference Δ*ϕ* is further analyzed in Supplementary Note 2.

### Parameters

All parameters used for numerical calculation can be found in Table 1. Only a few of them can be considered as free fitting parameters. In fact, the size of the Coulomb diamonds, the slopes of the resonant lines corresponding to transitions between ground states, and the height and broadening of the Coulomb oscillations allow us to fix most of them (i.e. *U*, *ε*_0_, *T*, *η* and the average tunneling rate (*Γ*_L_ + *Γ*_R_)/2). This analysis leaves four fitting parameters (*Γ*_L_/*Γ*_R_, Δ*ϕ*, *J* and Γ_rel_). However, *J* and Γ_rel_ are not sensitive parameters, as long as both are not much larger than the tunneling rates. The crucial fitting parameters are instead the difference of the tunneling phases Δ*ϕ* and the ratio *Γ*_L_/*Γ*_R_ of the tunneling rates. They control respectively the strength of the interference and the rectification in the *I* – *V* characteristics. Differently from the first set of parameters, the values of these last two were not extracted from experimental data, but rather tuned freely until convergence of the numerically calculated transport characteristics to the experimental ones.

## Supplementary information

Supplementary Information

## Data Availability

All relevant data are available from the authors.
