# Supplementary material for: Coherent population trapping by dark state formation in a carbon nanotube quantum dot
Source: Nat Commun. 2019 Jan 22;10:381. doi: 10.1038/s41467-018-08112-x (PMC6343009; doi:10.1038/s41467-018-08112-x)
Supplement: Supplementary file 1 — Supplementary Information [file 41467_2018_8112_MOESM1_ESM.pdf]

Supplementary Information

Coherent population trapping by dark state formation in a carbon nanotube quantum dot

A. Donarini et al.

SUPPLEMENTARY NOTE 1

EIGENSTATES AND DARK STATES OF A CARBON NANOTUBE

We discuss many-body configurations of a CNT quantum dot described by the Hamiltonian

$$\hat{H}_{\mathrm{CNT}} = \sum_{m\ell_z} m\varepsilon_0 \hat{n}_{m\ell_z} + \frac{U}{2}\hat{N}^2 + J\sum_m \left(\hat{\mathbf{S}}_{m\ell}\cdot\hat{\mathbf{S}}_{m-\ell} + \frac{1}{4}\hat{n}_{m\ell}\hat{n}_{m-\ell}\right), \tag{1}$$

also given in Eq. (5) in the main manuscript. Here, $m = 0, 1, 2$ runs over three longitudinal shells, ε_0 is the shell spacing, U accounts for charging effects, and J is the exchange interaction. The occupation operator $\hat{n}_{m\ell_z}$ defines the number of electrons in shell m with angular momentum ℓ_z, where a summation over the spin degree of freedom is performed. The eigenstates $|N, E; S, S_z, L_z\rangle$ of the CNT Hamiltonian are classified according to their occupation N with respect to a reference state $|0\rangle$ given below, their energy E and total spin and angular momentum quantum numbers S, S_z and L_z. The reference state $|0\rangle$ has the shell $m = 0$ completely full, and the upper two shells $m = 1, 2$ completely empty and corresponds to the $N = 0$ groundstate:

$$|0, E_0; 0, 0, 0\rangle \equiv |0\rangle = \quad . \tag{2}$$

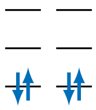

The left (right) states are for angular momentum values $\ell_z = +(-)\,\ell$ and the up/down arrows indicate opposite spin direction. The diagram in Eq. (2) fixes a set of occupations $\{n_{m\ell_z\sigma}\}$in the representation $|\{n_{m\ell_z\sigma}\}\rangle = \prod_{m\ell_z\sigma}(\hat{d}^\dagger_{m\ell_z\sigma})^{n_{m\ell_z\sigma}}|\emptyset\rangle$. Conventionally we fix the order of the creator operators such that electrons in the lower shells are created first. Moreover, within each shell we chose the ordering $d^\dagger_{m\ell\uparrow}d^\dagger_{m\ell\downarrow}d^\dagger_{m-\ell\uparrow}d^\dagger_{m-\ell\downarrow}$. We fix the energy of the state in Eq. (2) to be E_0. Many-body excited states with $N = 0$ are obtained by creating electron-hole pairs starting from this ground state configuration for fixed electron number. In Table 1 we show both the groundstate as well as all the first excited states, which have energy $\varepsilon_0 - J/2$ above the groundstate. The $N = 1$ groundstate is trivially four-fold degenerate

$$|1, E_{1_0} = \epsilon_0; \frac{1}{2}, \pm\frac{1}{2}, \pm\ell\rangle \equiv |\pm\frac{1}{2}, \pm\ell\rangle = \left\{ \quad , \quad , \quad , \quad \right\}, \tag{3}$$

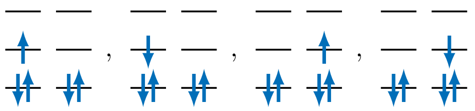

with these states playing a crucial role in the coherent population trapping (CPT) effect discussed in this work. In Tabs. 1, 2 the lowest excited states with $N = 1$, corresponding to the energies $\varepsilon_0 - J$, $\varepsilon_0 - J/2$, and ε_0 are also shown. Finally, in Tabs. 2, 3 we depict the lowest energy states with $N = 2, 3$. Notice that in the numerics we have considered only states up to a cut-off of $1.5\varepsilon_0$ above the respective groundstate.

From the many-body energies, addition energies $E^{\mathrm{add}}_N = \mu(N+1) - \mu(N) = E_{N+1} - 2E_N + E_{N-1}$ are easily calculated. We find $E^{\mathrm{add}}_0 = \varepsilon_0 + U - J/2$, $E^{\mathrm{add}}_1 = U - J/2$, $E^{\mathrm{add}}_2 = U + 3J/2$ and $E^{\mathrm{add}}_3 = U - J/2$ which in turn define the heights of the Coulomb diamonds.

A. Dark states for one electron

Eq. (3) allows one to construct linear combinations $|\mathrm{DS}, \sigma; \alpha\rangle$ of the single-particle groundstates $|\sigma, \ell_z\rangle$ which are decoupled at given positions $\mathbf{r}_\alpha$, and hence may act as dark states (DS) for a given bias polarity. Such states have the generic form $|\mathrm{DS}, \sigma; \alpha\rangle = a(\mathbf{r}_\alpha)|\sigma, \ell\rangle + b(\mathbf{r}_\alpha)|\sigma, -\ell\rangle$, where the coefficients satisfy the normalization condition $|a(\mathbf{r}_\alpha)|^2 + |b(\mathbf{r}_\alpha)|^2 = 1$ and are determined through the requirement

$$\langle 0|\hat{d}_{\alpha\sigma}|\mathrm{DS}, \sigma; \alpha\rangle \equiv 0, \tag{4}$$

where $\hat{d}_{\alpha\sigma}$ destroys a CNT electron of spin σ at position $\mathbf{r}_\alpha$. We express such operator in the angular momentum basis, $\hat{d}_{\alpha\sigma} = \sum_{m\ell_z}\langle\mathbf{r}_\alpha|m\ell_z\sigma\rangle\hat{d}_{m\ell_z\sigma}$, and observe that the orbital part $\phi_{m\ell_z\sigma}(\mathbf{r}_\alpha) = \langle\mathbf{r}_\alpha|m\ell_z\sigma\rangle$ of the CNT wave function is complex, $\phi_{m\ell_z\sigma}(\mathbf{r}_\alpha) = |\phi_{m\ell_z\sigma}(\mathbf{r}_\alpha)|e^{i\theta_\alpha(m,\ell_z)}$. Furthermore, $|\phi_{m\ell_z\sigma}(\mathbf{r}_\alpha)| = |\phi_{m-\ell_z-\sigma}(\mathbf{r}_\alpha)|$ due to time-reversal symmetry. Then, insertion in Eq. (4) yields for the coefficients the simple form $a(\mathbf{r}_\alpha) = e^{-i\theta_\alpha(m,\ell)}/\sqrt{2}$,

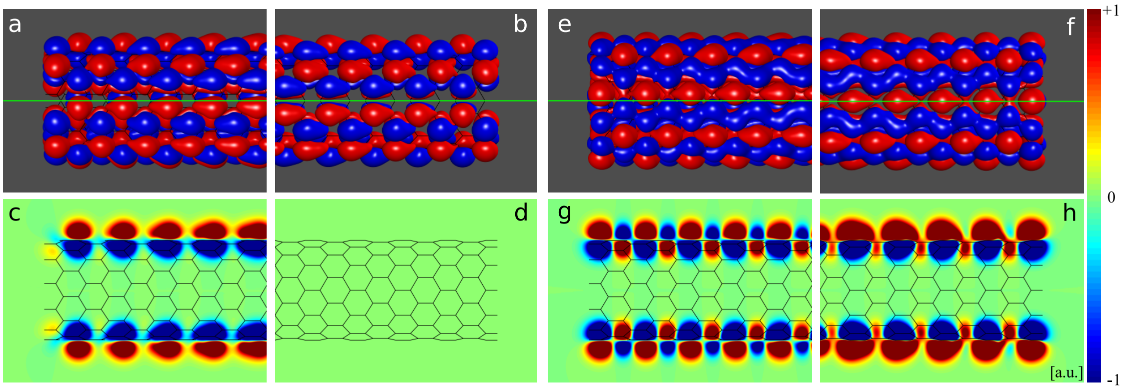

Fig. 1. Dark and coupled states of a (12,0) CNT. a,b Equiamplitude surface of the wave function associated to a decoupled state (DS) near the left (**a**) and right (**b**) ends. **c,d** Projection of the wave function on the intersection rectangle passing through the CNT and perpendiculat to the lead in the contact region (see Fig. 6a of the main text). **e-h** The same features discussed for the DS are here shown for its associated orthogonal coupled state (CS).

$b(\mathbf{r}_\alpha) = -e^{-i\theta_\alpha(m,-\ell)}/\sqrt{2}$. Introducing the angles $\bar{\theta}_\alpha = [\theta_\alpha(m,\ell)+\theta_\alpha(m,-\ell)]/2$, and $\Delta\theta_\alpha = [\theta_\alpha(m,\ell)-\theta_\alpha(m,-\ell)]/2$, we find - apart from an overall phase - for the DS the form given in Eq. (2) of the main text,

$$|\mathrm{DS}, \frac{1}{2}; \alpha\rangle = \frac{e^{-i\bar{\theta}_\alpha}}{\sqrt{2}} \left(e^{i\ell\phi_\alpha} \begin{array}{cc} - & - \\ \uparrow & - \\ \uparrow\downarrow & \uparrow\downarrow \end{array} - e^{-i\ell\phi_\alpha} \begin{array}{cc} - & - \\ - & \uparrow \\ \uparrow\downarrow & \uparrow\downarrow \end{array} \right),$$

$$|\mathrm{DS}, -\frac{1}{2}; \alpha\rangle = \frac{e^{-i\bar{\theta}_\alpha}}{\sqrt{2}} \left(e^{i\ell\phi_\alpha} \begin{array}{cc} - & - \\ \downarrow & - \\ \uparrow\downarrow & \uparrow\downarrow \end{array} - e^{-i\ell\phi_\alpha} \begin{array}{cc} - & - \\ - & \downarrow \\ \uparrow\downarrow & \uparrow\downarrow \end{array} \right), \quad (5)$$

where $\phi_\alpha \equiv -\Delta\theta_\alpha/\ell$. Given the form in Eq. (5) for the DS at lead α, one finds $\langle 0|\hat{d}_{\bar{\alpha}\sigma}|\mathrm{DS}, \sigma; \alpha\rangle \propto \sin(\ell(\phi_\mathrm{R} - \phi_\mathrm{L}))$ for the matrix element involving the destruction operator at the opposite lead $\bar{\alpha}$. Thus the requirement $\phi_\mathrm{R} \neq \phi_\mathrm{L}$ is necessary for a dark state to have vanishing transition amplitude only at one lead.

An example of dark and coupled states is shown in Fig. 1 for the case of a $(12,0)$ CNT discussed in the main text (see Figure 5 there). The CNT was chosen to be 100 unit cells long, corresponding to about 50nm. Moreover, the states shown correspond to the first excited state above the band gap.

B. Two-electrons dark states

The results of the $0 \leftrightarrow 1$ transitions can be easily extended to higher electron numbers since a dark state which blocks transitions to 2-electrons states can also be constructed. The exchange interaction in the CNT Hamiltonian in Eq. (5) with antiferromagnetic coupling J can be expressed as

$$\hat{H}_\mathrm{exch} = J \sum_m \left(\hat{\mathbf{S}}_{m\ell} \cdot \hat{\mathbf{S}}_{m-\ell} + \frac{1}{4}\hat{n}_{m\ell}\hat{n}_{m-\ell} \right) = -\frac{J}{2} \sum_{m\sigma\sigma'} \hat{d}^\dagger_{m\ell\sigma} \hat{d}^\dagger_{m\bar{\ell}\sigma'} \hat{d}_{m\ell\sigma'} \hat{d}_{m\bar{\ell}\sigma}, \quad (6)$$

which results in a spin-singlet ground-state

$$|2, E_{2_0} = 2\varepsilon_0 - \frac{J}{2}; 0, 0, 0\rangle = \frac{1}{\sqrt{2}} \left(\begin{array}{cc} - & - \\ \uparrow & \downarrow \\ \uparrow\downarrow & \uparrow\downarrow \end{array} - \begin{array}{cc} - & - \\ \downarrow & \uparrow \\ \uparrow\downarrow & \uparrow\downarrow \end{array} \right), \quad (7)$$

a doublet of angular momentum first excited states

$$|2, E_{2_1} = 2\varepsilon_0; 0, 0, 2\ell\rangle = \begin{array}{cc} - & - \\ \uparrow\downarrow & - \\ \uparrow\downarrow & \uparrow\downarrow \end{array}, \qquad |2, E_{2_1} = 2\varepsilon_0; 0, 0, -2\ell\rangle = \begin{array}{cc} - & - \\ - & \uparrow\downarrow \\ \uparrow\downarrow & \uparrow\downarrow \end{array}, \quad (8)$$

and a spin-triplet of second excited states with excitation energy $E_{2_2} - E_{2_0} = J$,

$$|2, E_{2_2}; 1, -1, 0\rangle = \begin{smallmatrix} \text{—} & \text{—} \\ \downarrow & \downarrow \\ \uparrow\downarrow & \uparrow\downarrow \end{smallmatrix} \;, \quad |2, E_{2_2}; 1, 0, 0\rangle = \frac{1}{\sqrt{2}} \left(\begin{smallmatrix} \text{—} & \text{—} \\ \uparrow & \downarrow \\ \uparrow\downarrow & \uparrow\downarrow \end{smallmatrix} + \begin{smallmatrix} \text{—} & \text{—} \\ \downarrow & \uparrow \\ \uparrow\downarrow & \uparrow\downarrow \end{smallmatrix} \right), \quad |2, E_{2_2}; 1, 1, 0\rangle = \begin{smallmatrix} \text{—} & \text{—} \\ \uparrow & \uparrow \\ \uparrow\downarrow & \uparrow\downarrow \end{smallmatrix} \;. \quad (9)$$

The one-particle dark states in Eq. (5) can block transitions to the two-electron ground-state. However, whether the blocking is effective crucially depends on the exchange energy J. In fact, as soon as the two-particles first excited doublet enters the transport window, interference is destroyed since transport through the doublet can occur. In our simulations we took indeed $J \simeq \Gamma = \Gamma_L + \Gamma_R$, such that the splitting of ground- and excited state is large enough to destroy interference at least partially and small enough to not see an additional excitation line appearing. Notice that in this situation the secular approximation breaks down, and the dynamics of the reduced density matrix is governed by a more general set of equations accounting also for nonsecular terms.[1]

Interestingly, for $J = 0$ (or at least $J \ll \Gamma_\alpha$), the two-particle ground state, which now is a sextuplet, can form a dark state itself

$$|2, \mathrm{DS}\rangle = \frac{1}{2} \left(e^{2i\ell\phi_\alpha} \begin{smallmatrix} \text{—} & \text{—} \\ \uparrow\downarrow & \text{—} \\ \uparrow\downarrow & \uparrow\downarrow \end{smallmatrix} - \begin{smallmatrix} \text{—} & \text{—} \\ \uparrow & \downarrow \\ \uparrow\downarrow & \uparrow\downarrow \end{smallmatrix} + \begin{smallmatrix} \text{—} & \text{—} \\ \downarrow & \uparrow \\ \uparrow\downarrow & \uparrow\downarrow \end{smallmatrix} + e^{-2i\ell\phi_\alpha} \begin{smallmatrix} \text{—} & \text{—} \\ \text{—} & \uparrow\downarrow \\ \uparrow\downarrow & \uparrow\downarrow \end{smallmatrix} \right), \quad (10)$$

which blocks transitions to the one-particle ground state at lead α since $\langle 1, E_{1_0}; \frac{1}{2}, \pm\frac{1}{2}, \pm\ell | \hat{d}_{\alpha\sigma} | 2, \mathrm{DS}\rangle = 0$. Again we require that this dark state is not completely decoupled from the dynamics and can be reached from the left lead $\langle 1, E_{1_0}; \frac{1}{2}, \pm\frac{1}{2}, \pm\ell | \hat{d}_{\bar{\alpha}\sigma} | 2, \mathrm{DS}\rangle \propto \sin(\ell(\phi_R - \phi_L)) \neq 0$.

SUPPLEMENTARY NOTE 2:

IMPACT OF PRECESSION, TEMPERATURE AND RELAXATION ON COHERENT POPULATION TRAPPING

In this section we give a closer look at the role played by the precession frequencies $\omega_{L/R}$, Eq. (11) of the Methods, on the shape of the current as a function of the bias voltage. Furthermore, we investigate the role of temperature and inelastic relaxation. We focus on the vicinity of the $0 \leftrightarrow 1$ resonance where, as seen from the comparison in Fig. 2b, the analytical expression for the current, Eq. (4) of the main text, well reproduces the numerics.

We first start with Figs. 2a, which shows the bias dependence of the numerically evaluated current for different temperatures. While the traditional step-like behavior of the current at positive bias for is temperature broadened via the Fermi function, the interference peak at negative bias is not. To understand this feature, we have depicted in Fig. 2c the precession frequencies $\omega_{L/R}$ in the same bias voltage range of panel (a) and for the same temperatures. It is clear that the current changes occur in the correspondence of changes in the peaks in the precession frequencies. In particular, the temperature basically only changes the height of the resonance peaks and leaves the tails invariant.

We observe that at fixed temperature the broadening of the precession frequencies peaks is largely dominated by the charging energy U, since $\omega_{L/R}$ vanish when the bias becomes of the order of U/e, as shown in Fig. 2d. Eq. (4) of the main text proofs that the current is dominated by a single precession frequency in the numerator, where the bias direction defines which one. If this precession frequency becomes zero, interference perfectly blocks the current. In the experiment U is so large that this behavior cannot be seen; excited states enter the bias window before this value of the bias voltage is reached.

In the main manuscript we have discussed two analytic expressions for the current, valid near the $0 \leftrightarrow 1$ resonance and corresponding to the case of vanishing relaxation rate, and vanishing phase difference, respectively. In principle Eq. (10) from the Methods can be solved fully analytically; however the resulting expression is very lengthy and not useful here. Therefore we display the full analytical current in Fig. 3 and compare it to the limiting cases. We show the dependence of the current on the phase difference $\Delta\phi$ for various values of the relaxation rate. As an example we choose a large negative bias with precession frequencies $\hbar\omega_L = 3\mu$eV and $\hbar\omega_R = 2\mu$eV. For low relaxation rates ($\Gamma_{\mathrm{rel}} \ll \Gamma_{L/R}$) the simple limit of Eq. (4) is recovered at $\Delta\phi \neq 0$. This agreement is expected since in the shown bias range excited states are far-off in energy. At $\Delta\phi = 0$ the current is always given by the incoherent limit from Eq. (13) of the Methods section.

SUPPLEMENTARY NOTE 3

TUNNELING RATE MATRIX

We observe that in some nanotube based devices the quantum dot region is defined electrostatically, and not, as it is assumed in this work, by the presence of tunneling barriers between the metallic contact and the CNT. Electrostatic confinement is characterized by a strong variation of the tunneling coupling and charging energy with the gate voltage, leading to different behaviors ranging from Fabry-Perot interference to Kondo physics and sequential tunneling as the gate is swept from negative to positive voltages. In our experiment we did not observe strong variations of the current as the gate voltage was swept across the band gap down to the deep n-doped regime considered here (11 v to 13 V), cfg. Fig. 4. This supports our assumption that the bottleneck for transport is given by the contact region between the metallic leads and the CNT.

In this section we want to show that, in general, the rate matrix defined in Eq. (7) of the Methods,

$$\mathit{\Gamma}^{m}_{\alpha\ell_z\ell_z'}(\mathit{\Delta}E) := \frac{2\pi}{\hbar}\sum_{\mathbf{k}} t^{*}_{\alpha\mathbf{k}m\ell_z} t_{\alpha\mathbf{k}m\ell_z'}\,\delta(\varepsilon_{\mathbf{k}} - \mathit{\Delta}E), \tag{11}$$

is non diagonal in the angular momentum basis. We then exemplify our considerations to the special case of a ring coupled to a metal, where some close analytical expressions for the rate matrix can be obtained.

A. Tunneling amplitude

We start from the tunneling Hamiltonian, whose form is given in Eq. (6) of the Methods

$$\hat{H}_{\rm tun} = \sum_{\alpha\mathbf{k}m\ell_z\sigma} t_{\alpha\mathbf{k}m\ell_z\sigma}\hat{d}^{\dagger}_{m\ell_z\sigma}\hat{c}_{\alpha\mathbf{k}\sigma} + \text{h.c.}. \tag{12}$$

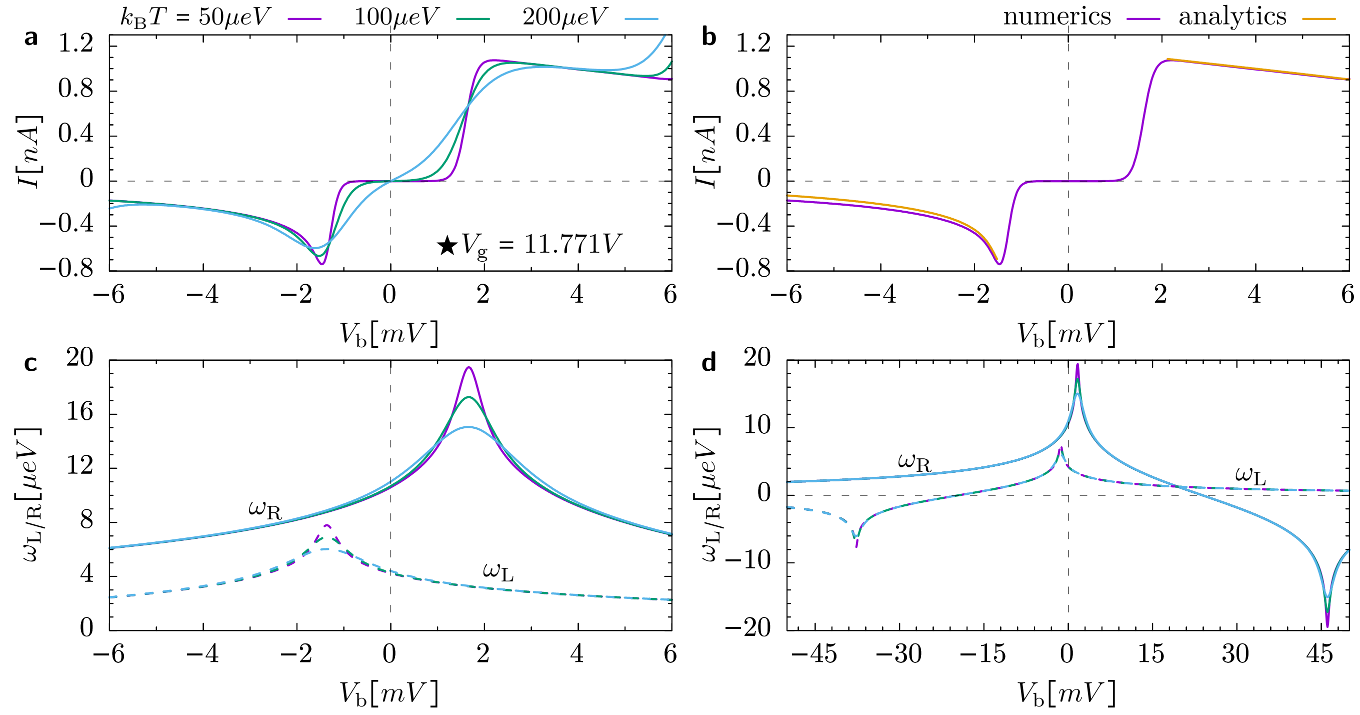

Fig. 2. **Bias dependence of current and precession frequencies. a** Numerical current bias traces around the $0 \leftrightarrow 1$ resonance at $V_{\rm g} = 11.771$V for different temperatures. **b** Comparison between the numerically calculated current and the analytic approximation valid for vanishing relaxation. The thermal energy was set to $k_{\rm B}T = 50\mu$eV. **c** Precession frequencies s $\omega_{\rm L/R}$ for the same temperatures as in (**a**). **d** Precession frequencies in a larger bias range. Their zeros occurs for energies $|eV_{\rm b}|$ of the order of the charging energy U.

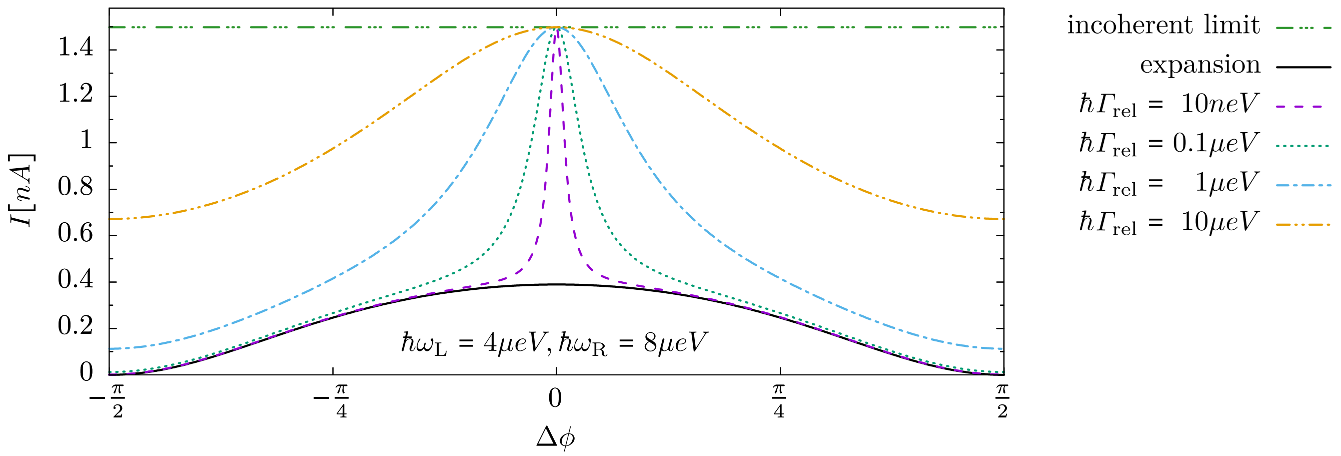

Fig. 3. Dependence of the current on the phase difference between left and right contacts. The numerical current is plotted for various values of the relaxation rate $\Gamma_{\rm rel}$. At low relaxation the current is well approximated by the analytical expression obtained for vanishing relaxation rate (black solid line). At large relaxation, the incoherent limit is approached where the current is independent of the phase difference. The precession frequencies are fixed at $\hbar\omega_{\rm L} = 4\mu$eV and $\hbar\omega_{\rm R} = 8\mu$eV. All other parameters are the same as in Tab. 1 given in the Methods section of the main manuscript.

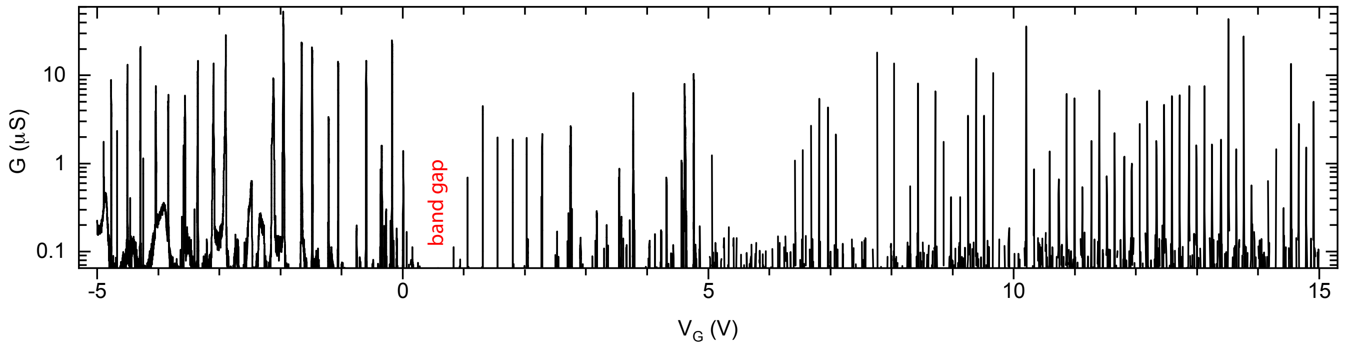

Fig. 4. Linear conductance gate sweep. The linear conductance over a large range of the gate voltage highlights the overall weak coupling of the CNT to the contacts.

The tunneling amplitude is proportional to the overlap of a wave function of the CNT $\phi_{m\ell_z\sigma}(\mathbf{r}) = \langle \mathbf{r}|m\ell_z\sigma\rangle$ and one of the lead $\psi_{\alpha\mathbf{k}\sigma}(\mathbf{r}) = \langle \mathbf{r}|\alpha\mathbf{k}\sigma\rangle$. Explicitly, $\langle \alpha\mathbf{k}\sigma|\hat{h}|m\ell_z\sigma'\rangle = t_{\alpha\mathbf{k}m\ell_z\sigma}\delta_{\sigma\sigma'}$, where $\hat{h} = \frac{\hat{p}^2}{2m_{\rm el}} + v(\hat{r})$ is the single-particle Hamiltonian of the CNT–leads complex. By decomposing the electrostatic potential into a contribution from the CNT and one from the leads, $v(\mathbf{r}) = v_{\rm CNT}(\mathbf{r}) + v_{\rm leads}(\mathbf{r})$, see the schematics in the Supplementary Figure 5, the tunneling amplitude can be written as

$$t_{\alpha\mathbf{k}m\ell_z\sigma} = \int \mathrm{d}\mathbf{r}\; \psi^*_{\alpha\mathbf{k}\sigma}(\mathbf{r})\left(\frac{\hat{p}^2}{2m_{\rm el}} + v(\mathbf{r})\right)\phi_{m\ell_z\sigma}(\mathbf{r}) = \langle \alpha\mathbf{k}\sigma|\hat{h}_{\rm CNT}|m\ell_z\sigma\rangle + \underbrace{\langle \alpha\mathbf{k}\sigma|v_{\rm leads}|m\ell_z\sigma\rangle}_{\approx 0} = \underbrace{m\varepsilon_0}_{=\varepsilon_m}\langle \alpha\mathbf{k}\sigma|m\ell_z\sigma\rangle, \quad (13)$$

where $\hat{h}_{\rm CNT}$ is the single particle part of the CNT Hamiltonian from Eq. (5) in the Methods section. Since the wave functions of the CNT are much more localized than the lead ones, the contribution containing the overlap of lead and CNT wave function in the lead region (where the potential $v_{\rm leads}$ is finite) can be neglected, yielding the simple expression

$$t_{\alpha\mathbf{k}m\ell_z\sigma} = \varepsilon_m \int \mathrm{d}\mathbf{r}\; \psi^*_{\alpha\mathbf{k}\sigma}(\mathbf{r})\phi_{m\ell_z\sigma}(\mathbf{r}). \quad (14)$$

Hence, the evaluation of the tunneling amplitude requires to take a closer look at the CNT wave functions as well as the lead wave functions in the tunneling region.

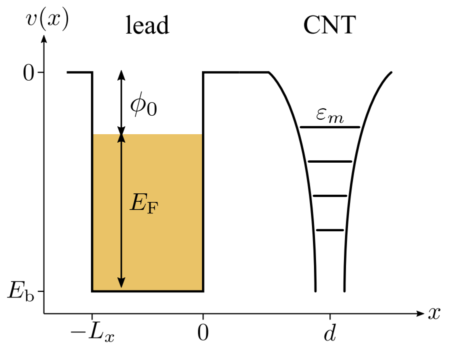

Fig. 5. **Potential landscape of the CNT-lead complex.** Electrostatic potential along the tunneling direction, chosen to be along the x-axis. In the lead, the electrons are considered to be free electrons in the direction parallel to the surface while they experience a confinement potential in the x-direction. $E_{\rm b}$ is the energy at the band bottom, $E_{\rm F}$ the Fermi energy and ϕ_0 the work function. Notice that the zero of the energy has been set to the vacuum. The CNT is located at a distance d from the lead and features localized bound states.

We start from the latter. We assume an adiabatically smooth variation of the lead surface in the contact region, such that the lead wave functions locally factorize in a contribution parallel to the surface and in an exponentially decaying part perpendicular to it:

$$\psi_{\alpha\mathbf{k}\sigma}(\mathbf{r}) = \psi^{\parallel}_{\alpha k_y k_z \sigma}(y,z)\psi^{\perp}_{\alpha k_x \sigma}(x) = \frac{1}{\sqrt{L_x}}\psi^{\parallel}_{\alpha k_y k_z \sigma}(y,z)e^{-\kappa_x x}. \tag{15}$$

Conservation of energy in the lead's potential well and in the tunneling region yields $E_{\rm el} = E_{\parallel} - \frac{\hbar^2\kappa_x^2}{2m_{\rm el}} = E_{\parallel} + E_b + \frac{\hbar^2 k_x^2}{2m_{\rm el}}$, where $E_{\rm el}$ is the energy of the lead electron with respect to the vacuum. Moreover, the energy at the band bottom is $E_{\rm b} = -(E_{\rm F} + \phi_0)$, with $E_{\rm F}$ the Fermi energy and ϕ_0 the lead work function, see the Supplementary Figure 5. Hence,

$$\kappa_x = \sqrt{\frac{2m_{\rm el}}{\hbar^2}\left(E_F^\alpha + \phi_0\right) - k_x^2}. \tag{16}$$

Thus, according to Eq. (16), the smallest values of κ_x, and hence the largest penetration in the CNT, are obtained when $k_x \approx k_F$ yielding $\kappa_x \approx \sqrt{2m_{\rm el}\phi_0/\hbar^2}$. Since the total energy is bound to be $E_{\rm F}^\alpha$, this simultaneously implies that the longitudinal components k_y, k_z should be vanishingly small (i.e. in the vicinity of the surface Γ–point).

Regarding the CNT wave functions, we assume that they are well described as a linear combination of atomic orbitals (LCAO) localized at the atomic positions $\mathbf{R}_j = (X_j, Y_j, Z_j)$. In particular, the low energy properties are already well captured by considering a single p-orbital for each atomic position.[2] We denote $|j\sigma\rangle$ such atomic state and the associated wave function as $p_\sigma(\mathbf{r} - \mathbf{R}_j) = \langle \mathbf{r}|j\sigma\rangle$. Hence, $|m\ell_z\sigma\rangle = \sum_{j\sigma} |j\sigma\rangle\langle j\sigma|m\ell_z\sigma\rangle = \sum_{j\sigma} |j\sigma\rangle c_j(m\ell_z\sigma)$, where the LCAO coefficients $c_j(m\ell_z\sigma) \equiv \langle j\sigma|m\ell_z\sigma\rangle$ have been introduced. Notice that they are chosen in such a way that the CNT wavefunctions obey proper boundary conditions at the ends of the tube.[3] Furthermore, due to time reversal symmetry, it holds $c_j(m\ell_z\sigma) = c_j^*(m - \ell_z - \sigma)$. It follows

$$t_{\alpha\mathbf{k}m\ell_z\sigma} = \varepsilon_m \sum_j c_j(m\ell_z\sigma)\int \mathrm{d}\mathbf{r}\; \psi^*_{\alpha\mathbf{k}\sigma}(\mathbf{r})p_\sigma(\mathbf{r} - \mathbf{R}_j). \tag{17}$$

In the *absence* of spin-orbit coupling, as in our case, the spatial and spin parts factorize both for the leads as well as the CNT wave functions, yielding spin-independent coefficients $c_j(m\ell_z\sigma) = c_j(m\ell_z)$. Similarly, the scalar product $\langle \alpha k\sigma|j\sigma\rangle$ becomes spin independent, yielding the final form for the tunneling amplitude

$$t_{\alpha\mathbf{k}m\ell_z\sigma} = t_{\alpha\mathbf{k}m\ell_z} = \varepsilon_m \sum_j c_j(m\ell_z)\int \mathrm{d}\mathbf{r}\; \psi^*_{\alpha\mathbf{k}}(\mathbf{r})p(\mathbf{r} - \mathbf{R}_j) \approx \varepsilon_m a \sum_j c_j(m\ell_z)\psi^*_{\alpha\mathbf{k}}(\mathbf{R}_j), \tag{18}$$

where in the last step we approximated the localized p-orbitals to Dirac-delta functions, $p(\mathbf{r}) = a\delta(\mathbf{r})$, centered at the atomic position $\mathbf{R}_j$. Here a is a normalization factor. The last approximation neglects the nodal plane of the p_z orbitals, but it is justified by i) the selection of j given by the lead wave function and ii) the negligible contribution to the integral given by the CNT wave function inside the tube.

B. Rate matrix of a CNT-metal complex

We calculate the rate matrix according to Eqs. (11) and (18), i.e., in the absence of spin-orbit coupling. We then obtain

$$\begin{aligned} \mathit{\Gamma}^m_{\alpha\ell_z\ell'_z}(\mathit{\Delta E}) &= \frac{2\pi}{\hbar}\varepsilon_m^2|a|^2\sum_{jj'}c_j^*(m\ell_z)c_{j'}(m\ell'_z)\sum_{\mathbf{k}}\psi_{\alpha\mathbf{k}}(\mathbf{R}_j)\psi^*_{\alpha\mathbf{k}}(\mathbf{R}_{j'})\delta(\varepsilon_{\mathbf{k}}-\mathit{\Delta E}) \\ &= \frac{2\pi}{\hbar}\frac{|a|^2}{L_x}\sum_{jj'}c_j^*(m\ell_z)c_{j'}(m\ell'_z)\sum_{\mathbf{k}}\psi^{\|}_{\alpha k_y k_z}(Y_j,Z_j)\psi^{\|*}_{\alpha k_y k_z}(Y_{j'},Z_{j'})e^{-\kappa_x(X_j+X_{j'})}\delta(\varepsilon_{\mathbf{k}}-\mathit{\Delta E}). \end{aligned} \tag{19}$$

Whether the rate matrix is diagonal in the angular momentum basis, crucially depends on the geometry of the contact region. The exponential $e^{-\kappa_x(X_j+X_{j'})}$ in fact selects in the sums over the atomic positions those CNT atoms closest to the leads. Furthermore, in the summation over the momenta $\mathbf{k}$, it selects the smallest values of κ_x compatible with the requirement that the energy of the tunneling lead electron is resonant with the CNT chemical potential $\mathit{\Delta E}$. As discussed in the previous subsection, this yields $k_y, k_z \approx 0$ and $\kappa_x \approx \sqrt{2m_{\mathrm{el}}\phi_0/\hbar^2} := \kappa_{\min}$, such that

$$\mathit{\Gamma}^m_{\alpha\ell_z\ell'_z}(\mathit{\Delta E}) \approx \frac{2\pi}{\hbar}\varepsilon_m^2\frac{|a|^2}{L_x}\sum_{jj'}c_j^*(m\ell_z)c_{j'}(m\ell'_z)e^{-\kappa_{\min}(X_j+X_{j'})}\psi^{\|}_{\alpha k_\|=0}(Y_j,Z_j)\psi^{\|*}_{\alpha k_\|=0}(Y_{j'},Z_{j'})\sum_{\mathbf{k}}\delta(\varepsilon_{\mathbf{k}}-\mathit{\Delta E}). \tag{20}$$

Thus, this so called surface Γ-point approximation[4] enables us to decouple the sums over j and j' into two independent sums. We introduce the density of states at the Fermi level $D = \sum_{\mathbf{k}}\delta(\varepsilon_{\mathbf{k}} - E_{\mathrm{F}})$ and the tunneling coefficients

$$\tau_\alpha(m\ell_z) = \varepsilon_m\frac{a}{\sqrt{L_x}}\sum_{j'}c_{j'}(m\ell_z)e^{-\kappa_{\min}X_{j'}}\psi^{\|*}_{\alpha k_\|=0}(Y_{j'},Z_{j'}), \tag{21}$$

yielding

$$\mathit{\Gamma}^m_{\alpha\ell_z\ell'_z}(\mathit{\Delta E}) = \frac{2\pi}{\hbar}D\tau^*_\alpha(m\ell_z)\tau_\alpha(m\ell'_z). \tag{22}$$

In the surface Γ-point approximation, the wave function $\psi^{\|}(Y_j,Z_j)$ is independent of k_y and k_z and hence real. Furthermore, the LCAO coefficients are related by time-reversal symmetry, $c_j(m,\ell_z) = c_j^*(m,-\ell_z)$, yielding the result $\tau_\alpha(m\ell_z) = \tau^*_\alpha(m-\ell_z)$. Accounting for this symmetry we finally obtain the final form for the rate matrix used in the main text

$$\mathit{\Gamma}^m_{\alpha\ell_z\ell'_z}(\mathit{\Delta E}) = \mathit{\Gamma}^m_\alpha e^{i\phi^m_\alpha(\ell_z-\ell'_z)}, \quad \mathit{\Gamma}^m_\alpha = \frac{2\pi}{\hbar}D|\tau_\alpha(m\ell_z)|^2, \quad \phi^m_\alpha := arg\{\tau_\alpha(m\ell)\}. \tag{23}$$

The result in Equation (23) strongly relies on the surface Γ-point approximation, which allows one to decouple the double sum over the atomic positions j and j'. In the next subsection we have explored the consequences of keeping a finite contribution for the parallel momenta k_y and k_z on the example of a ring of N carbon atoms coupled in three different ways to a metal. As we shall see, if the ring is lying flat on the substrate, such that all atoms are equally distant from the lead, the rate matrix becomes diagonal, see Supplementary Figure 6a. The result in Eq. (23) is in contrast recovered when the ring is orthogonal to the substrate, in a way that tunneling is dominated by only one closest atom, Supplementary Figure 6b. When two atoms are equally close to the surface, as for the case in figure 6c, the rate matrix is off-diagonal, but the modulus of the diagonal elements is smaller than that of the diagonal ones.

From this we conclude that in the CNT case, where only few atoms are close to the leads, the rate matrix is not diagonal. How good the simple form Eq. (23) describes the experiment, depends on various factors, among which tube's chirality. In the case of our experiment, we consider CNTs of the zig-zag class, which at the tube's end have non vanishing weights only for atoms of a given sublattice.[3] Thus, if at the left end only A atoms have non vanishing LCAO coefficients, this implies that the neighboring B atoms are not tunneling coupled, hence effectively achieving the situation described in the simple example in Fig. (6)b. At the right lead, the same considerations apply upon exchange of the role of atoms A and B.

C. Rate matrix of a ring-metal complex

In this subsection we calculate the rate matrix for a ring of N carbon atoms with radius R. To study the effects of the orientation of the ring with respect to the surface, we study three different configurations. In the first one, the

ring is lying parallel to the surface of the lead, at a distance d from it, as shown in the Supplementary Figure 6a; in the second one it is standing on the $x-y$ plane perpendicular to the lead plane like shown in Figure 6b; in the third one it is also standing but now in a way that two atoms are equally distant from the lead, as shown in Figure 6c. The rate matrix for the ring follows easily from the general expression Eq. (19) upon dropping the shell index m. Similar to the CNT, the ring has a C_N symmetry and its single particle eigenstates can be classified in terms of angular momentum, ℓ_z, and spin, σ, degrees of freedom. The LCAO coefficients follow from the diagonalization of the ring Hamiltonian, and have the form $c_j(\ell_z) = \langle j|\ell_z\rangle = \frac{1}{\sqrt{N}} e^{i\frac{2\pi}{N} j\ell_z}$. Eq. (19) yields then for the ring the rate matrix

$$\Gamma_{\alpha\ell_z\ell'_z}(\Delta E) = \frac{2\pi}{\hbar}\frac{\varepsilon^2}{N}|a|^2 \sum_{jj'} e^{-i\frac{2\pi}{N}(j\ell_z - j'\ell'_z)} \sum_{\mathbf{k}} \psi_{\alpha\mathbf{k}}(\mathbf{R}_j)\psi^*_{\alpha\mathbf{k}}(\mathbf{R}_{j'})\delta(\varepsilon_{\mathbf{k}} - \Delta E) \tag{24}$$

$$= \frac{2\pi}{\hbar}\frac{\varepsilon^2}{N}\frac{|a|^2}{L_x} \sum_{jj'} e^{-i\frac{2\pi}{N}(j\ell_z - j'\ell'_z)} \sum_{\mathbf{k}} \psi^{\parallel}_{\alpha\mathbf{k}_\parallel}(\mathbf{R}_j)\psi^{\parallel *}_{\alpha\mathbf{k}_\parallel}(\mathbf{R}_{j'}) e^{-\kappa_x(X_j + X_{j'})}\delta(\varepsilon_{\mathbf{k}} - \Delta E). \tag{25}$$

To further simplify the calculation we consider in the following a plane wave behavior for the parallel wave function, $\psi^{\parallel}_{\alpha\mathbf{k}_\parallel}(\mathbf{R}) = \frac{1}{\sqrt{S}} e^{i\mathbf{k}_\parallel\cdot\mathbf{R}}$, with S a normalization constant.

Case a

Let us consider the first case where the ring is lying planar on top of the lead at a distance $X_j = d$. The rate matrix then reads

$$\Gamma_{\alpha\ell_z\ell'_z}(\Delta E) = \frac{2\pi}{\hbar}\frac{\mathcal{C}\varepsilon^2}{N} \sum_{jj'} e^{-i\frac{2\pi}{N}(j\ell_z - j'\ell'_z)} \sum_{\mathbf{k}} e^{i\mathbf{k}_\parallel\cdot(\mathbf{R}_j - \mathbf{R}_{j'})} e^{-2\kappa_x d}\delta(\epsilon_{\mathbf{k}} - \Delta E), \tag{26}$$

where $\mathcal{C} =: |a|^2/(SL_x)$. It is convenient to express $j' = j' - j + j := -\Delta j + j$ and to observe that $|\mathbf{R}_j - \mathbf{R}_{j'}| := R_{\Delta j}$ only depends on the relative distance Δj but not on the position j. This suggests to transform the sum over momentum into an integral, and to express this integral in cylindrical coordinates $k_y, k_z \to \varphi, k_\parallel$. This results in

$$\Gamma_{\alpha\ell_z\ell'_z}(\Delta E) = \frac{2\pi}{\hbar}\frac{\mathcal{C}\varepsilon^2}{N} \overbrace{\sum_{j} e^{-i\frac{2\pi}{N} j(\ell_z - \ell'_z)}}^{N\delta_{\ell_z\ell'_z}} \sum_{\Delta j} e^{-i\frac{2\pi}{N}\Delta j \ell'_z} \int \mathrm{d}\varphi \int \mathrm{d}k_\parallel \int \mathrm{d}k_x e^{ik_\parallel R_{\Delta j}\cos\varphi} k_\parallel e^{-2\kappa_x d}\delta(\varepsilon_{\mathbf{k}} - \Delta E). \tag{27}$$

The integration over the angle φ results in a real function of $k_\parallel$; similarly, in the sum over Δj for each finite positive Δj there is a negative counterpart. This leaves us with the diagonal and real rate matrix

$$\mathbf{\Gamma}_\alpha = \Gamma_\alpha \begin{pmatrix} 1 & 0 \\ 0 & 1 \end{pmatrix}, \tag{28}$$

which therefore does not support dark states. The absolute value of the diagonal parts is not important in this consideration. For simplicity and homogeneity with the other cases we have kept the δ approximation for the p_z functions. The description of a CNT "slice" would rather require to consider the orbital structure of the radially distributed p orbitals. Eq. (28) is obtained, though, also out of more fundamental symmetry arguments:

$$\Gamma_{\alpha\ell_z\ell'_z} = \frac{2\pi}{\hbar}\varepsilon_m^2 \sum_{\mathbf{k}} \langle m\ell_z|\alpha\mathbf{k}\rangle\langle\alpha\mathbf{k}|m\ell'_z\rangle = \frac{2\pi}{\hbar}\varepsilon_m^2 \sum_{\mathbf{k}} \langle m\ell_z|\hat{C}_N^\dagger \hat{C}_N|\alpha\mathbf{k}\rangle\langle\alpha\mathbf{k}|\hat{C}_N^\dagger \hat{C}_N|m\ell'_z\rangle$$

$$= \frac{2\pi}{\hbar}\varepsilon_m^2 e^{i\frac{2\pi}{N}(\ell'_z - \ell_z)} \sum_{\mathbf{k}} \langle m\ell_z|\alpha\mathbf{k}\rangle\langle\alpha\mathbf{k}|m\ell'_z\rangle = e^{i\frac{2\pi}{N}(\ell'_z - \ell_z)}\Gamma_{\alpha\ell_z\ell'_z}, \tag{29}$$

where $\hat{C}_N$ is the rotation of $2\pi/N$ around the x axis and the isotropy of the leads is assumed. Eq. (29) implies $\mathbf{\Gamma}_\alpha$ is diagonal. The form in Eq. (28) follows by requiring time reversal symmetry.

Case b

In the second case the result is quite different. The rate matrix for the standing ring is

$$\Gamma_{\alpha\ell_z\ell'_z}(\Delta E) = \frac{2\pi}{\hbar}\frac{\mathcal{C}\varepsilon^2}{N} \sum_{jj'} e^{-i\frac{2\pi}{N}(j\ell_z - j'\ell'_z)} \sum_{\mathbf{k}} e^{ik_y(Y_j - Y_{j'}) - \kappa_x(X_j + X_{j'})}\delta(\varepsilon_{\mathbf{k}} - \Delta E), \tag{30}$$

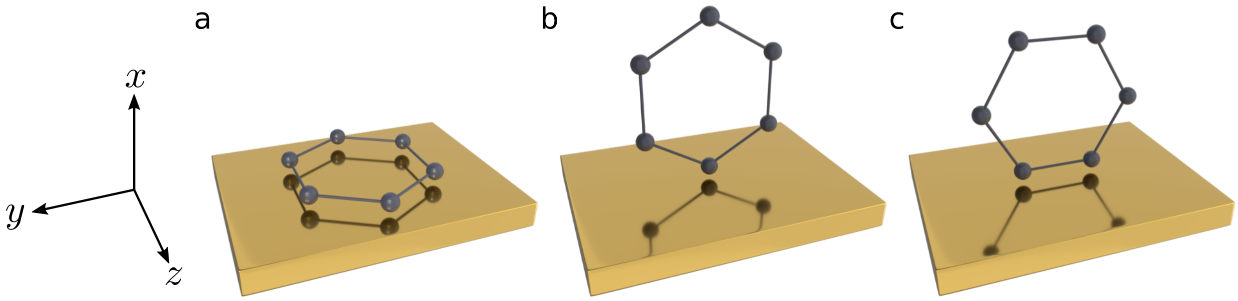

Fig. 6. **Tunneling configurations for a ring-lead complex.** **a** The ring lies flat on the lead surface. All atoms of the ring are equidistant from the surface. **b** The ring is standing perpendicular to the lead with only one carbon atom closest to the surface. **c** Similar to the previous case, the ring is standing perpendicular to the surface but rotated such that two carbon atoms are equidistant from the lead.

where $\mathcal{C} = |a|^2/(SL_x)$. One can see immediately that the trick used in the previous case a does not work here, due to the dependence on j (j') of the variables X_j (X'_j). An estimation of $\kappa_x = \sqrt{\frac{2m_{\rm el}}{\hbar^2}\left(E_F^\alpha + \phi_0^\alpha\right) - k_x^2} \geq \sqrt{\frac{2m_{\rm el}}{\hbar^2}\phi_0^\alpha} = \mathcal{O}(\text{Å}^{-1})$ for typical work functions $\phi_0 = \mathcal{O}(\text{eV})$ tells that the contribution to the rate matrix shrinks by one order of magnitude for a distance of 1Å of the atom to the lead surface. This suggests that perfect local tunneling to the atom closest to the lead $j = j' = J$ at distance $X_J = d$ is a good approximation. We then obtain

$$\Gamma_{\alpha\ell_z\ell'_z} = \frac{2\pi}{\hbar}\frac{\mathcal{C}\varepsilon^2}{N} e^{-iJ\frac{2\pi}{N}(\ell_z-\ell'_z)} \sum_{\mathbf{k}} e^{-2\kappa_x^\alpha d}\delta(\epsilon_{\mathbf{k}} - \Delta E) \qquad \Rightarrow \qquad \mathbf{\Gamma}_\alpha = \Gamma_\alpha \begin{pmatrix} 1 & e^{2i\phi_\alpha} \\ e^{-2i\phi_\alpha} & 1 \end{pmatrix}. \tag{31}$$

Thus the single atom contact yields a rate matrix with maximal coherence, like in the surface Γ-point approximation discussed in the previous subsection.

Case c

In the third case the ring is rotated in a way that tunneling can occur through two atoms J and J' which are both in contact with the lead ($X_J = X_{J'} = d$). The rate matrix reads

$$\Gamma_{\alpha\ell_z\ell'_z}(\Delta E) = \frac{2\pi}{\hbar}\frac{\mathcal{C}\varepsilon^2}{N} \sum_{jj'\in(J,J')} e^{-i\frac{2\pi}{N}(j\ell_z - j'\ell'_z)} \sum_{\mathbf{k}} e^{ik_y(Y_j - Y_{j'}) - 2\kappa_x d}\delta(\epsilon_{\mathbf{k}} - \Delta E). \tag{32}$$

The diagonal and off-diagonal elements of the rate matrix can be simplified to

$$\Gamma_{\alpha\ell\ell}(\Delta E) = \frac{4\pi}{\hbar}\frac{\mathcal{C}\varepsilon^2}{N} \sum_{\mathbf{k}} \left[1 + \cos\left(\frac{2\pi}{N}\ell(J - J')\right)\cos\left(k_y \Delta Y\right)\right] e^{-2\kappa_x d}\delta(\epsilon_{\mathbf{k}} - \Delta E), \tag{33}$$

$$\Gamma_{\alpha\ell-\ell}(\Delta E) = \frac{4\pi}{\hbar}\frac{\mathcal{C}\varepsilon^2}{N} e^{-i\frac{2\pi}{N}\ell(J+J')} \sum_{\mathbf{k}} \left[\cos\left(\frac{2\pi}{N}\ell(J - J')\right) + \cos\left(k_y \Delta Y\right)\right] e^{-2\kappa_x d}\delta(\epsilon_{\mathbf{k}} - \Delta E), \tag{34}$$

with $\Delta Y = Y_J - Y_{J'}$. We used the fact that the sum over $\mathbf{k}$ is isotropic and therefore $\sin(k_y \Delta Y) \to 0$. One can see directly that for $\Delta Y = 0$ the result of case **b** is recovered. For $\Delta Y \neq 0$ the amplitude of the off-diagonal terms in $\mathbf{\Gamma}_\alpha$ is smaller than the diagonal values since in general $1 + \cos x \cos y - \cos x - \cos y = (\cos x - 1)(\cos y - 1) \geq 0$. We obtain a rate matrix intermediate to cases **a** and **b**

$$\mathbf{\Gamma}_\alpha = \Gamma_\alpha \begin{pmatrix} 1 & he^{2i\phi_\alpha} \\ he^{-2i\phi_\alpha} & 1 \end{pmatrix}, \quad 0 \leq h \leq 1. \tag{35}$$

SUPPLEMENTARY REFERENCES

[1] D. Darau, G. Begemann, A. Donarini, and M. Grifoni, Phys. Rev. B **79**, 235404 (2009).
[2] M. Dresselhaus, G. Dresselhaus, and A. Jorio, *Group Theory: Application to the Physics of Condensed Matter* (Springer Berlin Heidelberg, 2007).
[3] M. Marganska, P. Chudzinski, and M. Grifoni, Phys. Rev. B **92**, 075433 (2015).
[4] J. Tersoff and D. R. Hamann, Phys. Rev. B **31**, 805 (1985).

$4N$

ΔE	S	S_z	L_z	Eigenstate
0	0	0	0	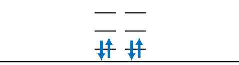
$\varepsilon_0 - \frac{J}{2}$	0	0	0	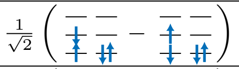
				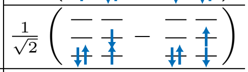
	1	1	0	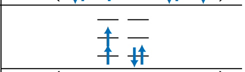
		0		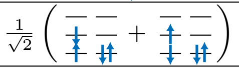
		-1		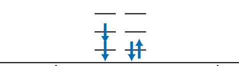
	0	0	2ℓ	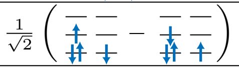
	1	1	2ℓ	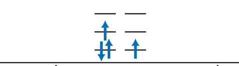
		0		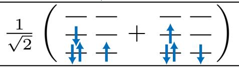
		-1		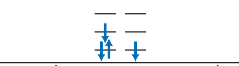
	0	0	-2ℓ	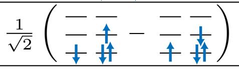
	1	1	-2ℓ	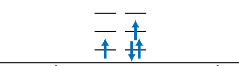
		0		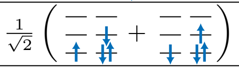
		-1		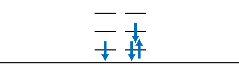

$4N+1$

ΔE	S	S_z	L_z	Eigenstate
0	$\frac{1}{2}$	$\frac{1}{2}$	ℓ	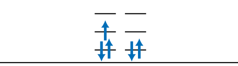
		$-\frac{1}{2}$		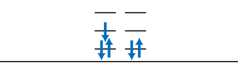
	$\frac{1}{2}$	$\frac{1}{2}$	$-\ell$	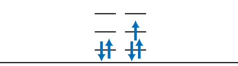
		$-\frac{1}{2}$		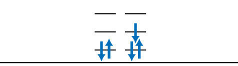
$\varepsilon_0 - J$	$\frac{1}{2}$	$\frac{1}{2}$	ℓ	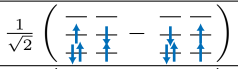
		$-\frac{1}{2}$		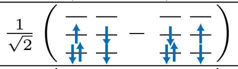
	$\frac{1}{2}$	$\frac{1}{2}$	$-\ell$	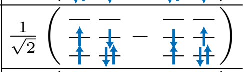
		$-\frac{1}{2}$		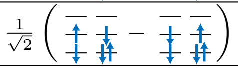
$\varepsilon_0 - \frac{J}{2}$	$\frac{1}{2}$	$\frac{1}{2}$	3ℓ	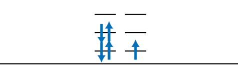
		$-\frac{1}{2}$		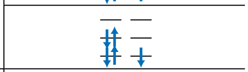
	$\frac{1}{2}$	$\frac{1}{2}$	ℓ	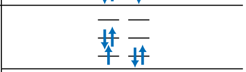
		$-\frac{1}{2}$		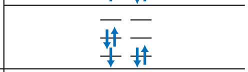
	$\frac{1}{2}$	$\frac{1}{2}$	$-\ell$	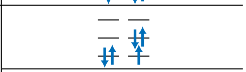
		$-\frac{1}{2}$		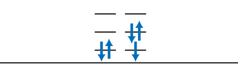
	$\frac{1}{2}$	$\frac{1}{2}$	-3ℓ	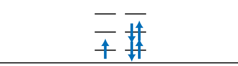
		$-\frac{1}{2}$		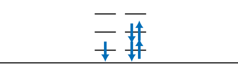

Tab. 1. **Eigenstates of a CNT for N=0,1.** First few eigenstates of a three shell CNT Hamiltonian with $N = 0$ (left) and $N = 1$ (right) electrons above the reference state. ΔE is the energy above the ground states with energies E_{4Ng} and $E_{4N+1g} = E_{4Ng} + \varepsilon_0 + U/2$.

$4N+1$

ΔE	S	S_z	L_z
ε_0	$\frac{1}{2}$	$\frac{1}{2}$	ℓ
		$-\frac{1}{2}$	
	$\frac{1}{2}$	$\frac{1}{2}$	$-\ell$
		$-\frac{1}{2}$	
	$\frac{3}{2}$	$\frac{3}{2}$	ℓ
		$\frac{1}{2}$	
		$-\frac{1}{2}$	
		$-\frac{3}{2}$	
	$\frac{3}{2}$	$\frac{3}{2}$	$-\ell$
		$\frac{1}{2}$	
		$-\frac{1}{2}$	
		$-\frac{3}{2}$	

Eigenstate

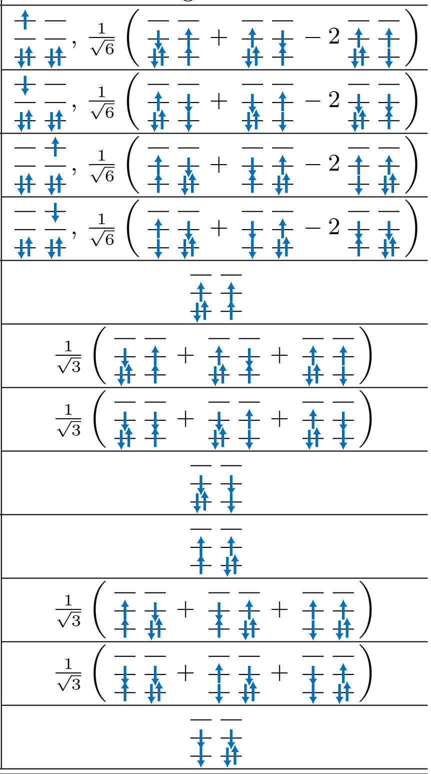

$4N+2$

ΔE	S	S_z	L_z
0	0	0	0
$\frac{J}{2}$	0	0	2ℓ
			-2ℓ
J	1	1	0
		0	
		-1	
$\varepsilon_0+\frac{J}{2}$	0	0	2ℓ
	1	1	2ℓ
		0	
		-1	
	0	0	0
	1	1	0
		0	
		-1	
	0	0	-2ℓ
	1	1	-2ℓ
		0	
		-1	

Eigenstate

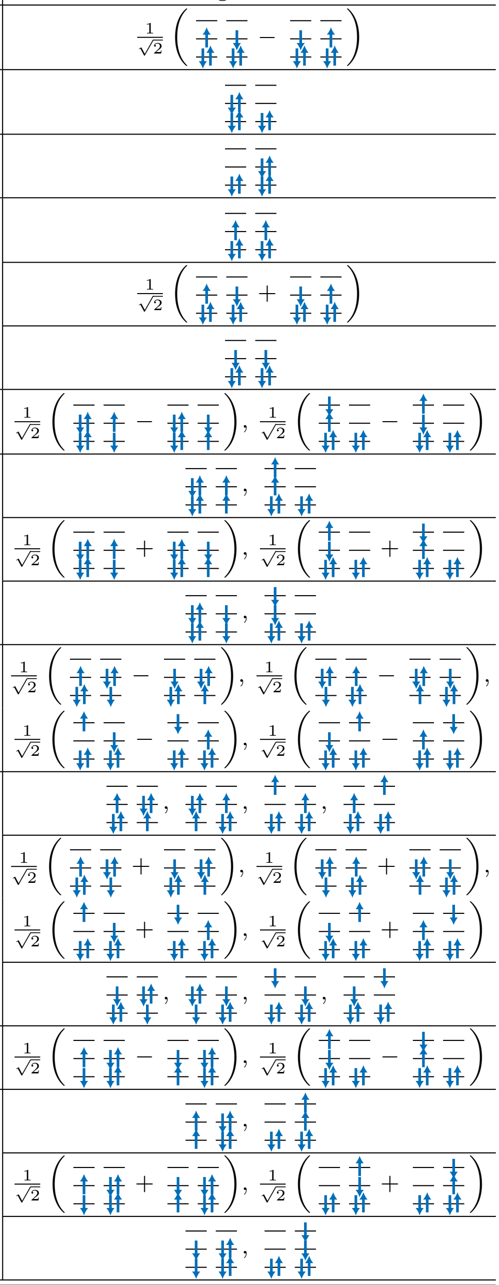

Tab. 2. **Eigenstates of a CNT for N=1,2.** Next few eigenstates of a three shell CNT Hamiltonian with $N=1$ (left) electrons above the reference state and the first few eigenstates with $N=2$ (right). ΔE is the energy above the ground states with energies $E_{4N+1g}=E_{4Ng}+\varepsilon_0+U/2$ and $E_{4N+2g}=E_{4Ng}+2\varepsilon_0+2U-J/2$.

$4N+3$

ΔE	S	S_z	L_z	Eigenstate
0	$\frac{1}{2}$	$\frac{1}{2}$	ℓ	
		$-\frac{1}{2}$		
	$\frac{1}{2}$	$\frac{1}{2}$	$-\ell$	
		$-\frac{1}{2}$		
$\varepsilon_0 - J$	$\frac{1}{2}$	$\frac{1}{2}$	ℓ	$\frac{1}{\sqrt{2}}$ (−)
		$-\frac{1}{2}$		$\frac{1}{\sqrt{2}}$ (−)
	$\frac{1}{2}$	$\frac{1}{2}$	$-\ell$	$\frac{1}{\sqrt{2}}$ (−)
		$-\frac{1}{2}$		$\frac{1}{\sqrt{2}}$ (−)
$\varepsilon_0 - \frac{J}{2}$	$\frac{1}{2}$	$\frac{1}{2}$	3ℓ	
		$-\frac{1}{2}$		
	$\frac{1}{2}$	$\frac{1}{2}$	ℓ	
		$-\frac{1}{2}$		
	$\frac{1}{2}$	$\frac{1}{2}$	$-\ell$	
		$-\frac{1}{2}$		
	$\frac{1}{2}$	$\frac{1}{2}$	-3ℓ	
		$-\frac{1}{2}$		

$4N+3$

ΔE	S	S_z	L_z	Eigenstate
ε_0	$\frac{1}{2}$	$\frac{1}{2}$	ℓ	, $\frac{1}{\sqrt{6}}$ (+ − 2)
		$-\frac{1}{2}$		, $\frac{1}{\sqrt{6}}$ (+ − 2)
	$\frac{1}{2}$	$\frac{1}{2}$	$-\ell$	, $\frac{1}{\sqrt{6}}$ (+ − 2)
		$-\frac{1}{2}$		, $\frac{1}{\sqrt{6}}$ (+ − 2)
	$\frac{3}{2}$	$\frac{3}{2}$	ℓ	
		$\frac{1}{2}$		$\frac{1}{\sqrt{3}}$ (+ +)
		$-\frac{1}{2}$		$\frac{1}{\sqrt{3}}$ (+ +)
		$-\frac{3}{2}$		
	$\frac{3}{2}$	$\frac{3}{2}$	$-\ell$	
		$\frac{1}{2}$		$\frac{1}{\sqrt{3}}$ (+ +)
		$-\frac{1}{2}$		$\frac{1}{\sqrt{3}}$ (+ +)
		$-\frac{3}{2}$		

Tab. 3. Eigenstates of a CNT for N=3. First few eigenstates of a three shell CNT Hamiltonian with $N = 3$ electrons above the reference state. ΔE is the energy above the ground state with energy $E_{4N+3g} = E_{4Ng} + 3\varepsilon_0 + 9U/2 + J/2$.
